# Optimized green zinc oxide/chitosan/amoxicillin nanocomposite against *Pseudomonas aeruginosa*

**DOI:** 10.1007/s00253-026-13768-3

**Published:** 2026-03-24

**Authors:** Mohamed M. El-Zahed, Yassmein M. Radwan, Mohamed I. Abou-Dobara

**Affiliations:** Department of Botany and Microbiology, Faculty of Science, Damietta University, New Damietta, 34517 Egypt

**Keywords:** Zinc oxide, RSM optimization, Nanocomposite, Anti-*Pseudomonas aeruginosa*, Antibiofilm

## Abstract

**Abstract:**

Multidrug-resistant *Pseudomonas aeruginosa* poses a significant global health threat, highlighting the need for innovative antimicrobial strategies. This study presents the successful synthesis, optimization, and characterization of a novel zinc oxide/chitosan/amoxicillin (ZnO/CS/AMX) nanocomposite aimed at combating *P. aeruginosa* infections, including the ability to form biofilms. Response surface methodology (RSM) based on the Box-Behnken design (BBD) was used to optimize the production of ZnO NPs using cell-free metabolites of *P. aeruginosa*. The optimal parameters for biosynthesis of ZnO NPs require a mixing ratio of 1:4 v/v% between the cell-free bacterial metabolites and 30 mM Zn precursor (Zn(NO_3_)_2_.6H_2_O) at pH 7.0 and 30 °C. The nanocomposite was synthesized by encapsulating amoxicillin (AMX) within chitosan-coated zinc oxide nanoparticles (ZnO NPs). Its properties were confirmed through various characterization techniques including UV–Vis spectroscopy (340 to 380 nm), XRD (101 plane, an average crystallite size = 59 nm), FTIR (Zn–O and proteins stretching vibrations), TEM (spherical to quasi-spherical in shape with size range = 36–98 nm), and zeta potential analyses (+ 35 ± 2.3 mV). The maximum drug loading of AMX in the ZnO/CS/AMX nanocomposite was found to be 55.7%. The antimicrobial effectiveness was thoroughly assessed against various clinical isolates of *P. aeruginosa*, all of which showed natural resistance to chitosan (CS) and AMX individually, as well as the reference strain ATCC 27853. Quantitative assays further confirmed the superior bactericidal potential of the nanocomposite compared to ZnO NPs and imipenem. The nanocomposite achieved exceptionally low minimum inhibitory concentration (MIC) and minimum bactericidal concentration (MBC) levels, as low as 10 µg/ml against resistant clinical isolates. TEM micrographs of *P. aeruginosa* cells treated with the nanocomposite revealed severe cellular damage. This damage included extensive separation between the cell wall and cytoplasmic membrane, complete cell lysis, and severe malformations. In addition, the nanocomposite exhibited outstanding antibiofilm activity at concentrations as low as 50 µg/ml. This activity dramatically increased in a dose-dependent manner, achieving ≤ 100% biofilm inhibition at 150 µg/ml. The cytotoxicity assessment on Vero cells demonstrated a promising safety profile, with a CC_50_ of 292 ± 1.3 µg/ml for the nanocomposite, nearly three times higher than that of ZnO NPs (106 ± 0.9 µg/ml). This wide therapeutic window indicates that ZnO/CS/AMX can effectively combat *P. aeruginosa* at concentrations far below that toxic to mammalian cells. These findings demonstrate that the ZnO/CS/AMX nanocomposite acts through a synergistic, multimodal mechanism, effectively overcoming bacterial resistance and biofilm recalcitrance, while also exhibiting favorable biocompatibility.

**Key points:**

- *RSM–Box–Behnken optimized green biosynthesis of ZnO NPs using cell-free metabolites of P. aeruginosa*
- *Green synthesis of zinc oxide/chitosan/amoxicillin nanocomposite to combat MDR P. aeruginosa*
- *The nanocomposite markedly enhances bactericidal and antibiofilm efficacy at low doses with high mammalian cell safety and synergistic action*

**Supplementary Information:**

The online version contains supplementary material available at 10.1007/s00253-026-13768-3.

## Introduction

Antimicrobial resistance (AMR) has emerged as one of the most significant global issues of the twenty-first century due to the rapid increase in AMR infection rates and the insufficient development of new antimicrobial drugs to combat this issue (Murray et al. 2022; Tang et al. 2023). AMR is predicted to become the world’s biggest cause of death by 2050 if preventive measures are not taken (O’Neill J 2016). According to estimates, over 1.2 million deaths worldwide were directly linked to antimicrobial resistance (AMR) in 2019. If insufficient steps are taken to reduce AMR, predictions indicate that the number could rise to about 10 million deaths per year by 2050 (Adamie et al. 2024). 

Self-medication, improper prescriptions, and inadequate therapies are common causes of excessive antibiotic use, leading to bacterial resistance. The demand for antibiotics increases with the prevalence of infectious diseases, which are often exacerbated by sanitation and hygiene practices. The ineffectiveness of low-quality drugs in delivering adequate levels of active ingredients can worsen resistance. Microorganisms have developed various defense mechanisms, such as structural changes and unique metabolic pathways, to avoid or counteract antimicrobial drugs (Ahmed et al. 2024).

Many bacterial resistance mechanisms, such as decreased membrane permeability, the use of efflux pumps, enzymatic modification or degradation of antibiotics, modification of antibiotic targets, and adaptive strategies like biofilm formation, reduce the effectiveness of almost all known antibiotics. AMR often appears within 0–6 months after drug use, underscoring the serious issue that AMR presents. It is important to note that the time it takes for resistance to develop can vary depending on the specific antibiotic-bacteria combination (Poku et al. 2022).

According to the 2022 Global Burden of Disease research, *Escherichia coli*, *Klebsiella pneumoniae*, and *Pseudomonas aeruginosa* were responsible for approximately 70% of antimicrobial resistance-related deaths in 2019 (Miller and Arias 2024). *P. aeruginosa*, a gram-negative opportunistic bacterium, is one of the powerful bacterial pathogens causing this crisis. It is primarily responsible for serious and often fatal healthcare-associated infections (HAIs) (Sandu et al. 2025). This adaptable bacterium is associated with a wide range of clinical pathologies, including burn wound infections, urinary tract infections (UTIs), bloodstream infections, ventilator-associated pneumonia, and chronic conditions in immunocompromised individuals and patients with cystic fibrosis (Litwin et al. 2021). The remarkable ability of *P. aeruginosa* to develop resistance to multiple classes of antibiotics, leading to the emergence of multidrug-resistant (MDR) and extensively drug-resistant (XDR) strains, is a defining feature of its virulence and innate resistance to therapeutic agents (Mirzaei et al. 2020). These resistant phenotypes severely restrict treatment options, leading to longer hospital stays, higher healthcare costs, and increased patient mortality.

In addition to intrinsic and acquired mechanisms of antibiotic resistance, such as efflux pumps, enzymatic inactivation, and porin channel modifications, *P. aeruginosa*’s proficiency in forming robust biofilms presents an additional layer of therapeutic complexity (Yin et al. 2022). A self-secreted extracellular polymeric substance (EPS) matrix envelops organized microbial populations called biofilms. This matrix serves as a protective barrier by preventing antibiotic penetration and creating a confined environment where bacteria show markedly increased tolerance to antimicrobial chemicals and host immunological responses (Wang et al. 2023). A significant clinical challenge that contributes to the chronicity and recurrence of infections is the efficient removal of developed *P. aeruginosa* biofilms. There is an urgent need to investigate novel and synergistic antimicrobial treatments due to the diminishing pipeline of conventional antibiotics and the rising incidence of MDR and biofilm-associated strains.

Nanoparticles (NPs) can be used as antimicrobials on their own or in conjunction with antibiotics to address the issue of AMR. The size of NPs ranges from 1 to 100 nm, and they possess limited disease resistance, minimal toxicity, antifungal properties, and chemical stability (Manimaran et al. 2023; Govindasamy et al. 2024a). NPs, such as Ag, Cu, Au, Zn, Fe, and Se, play a crucial role in agriculture, food, the environment, and nanomedicine (Indumathi et al. 2024; Falemban et al. 2025; Ramesh et al. 2025). There are three methods for creating NPs: chemical, biological, and physical. Environmentally friendly nanoparticle production has become more popular due to the increasing costs and environmental risks associated with physical and chemical processes. The product includes bacteria, fungus, and plant extracts. By reducing the use of harmful chemicals and improving nanoparticle biocompatibility, this eco-friendly method offers a sustainable alternative to traditional synthesis methods (Khan et al. 2022a). There are two types of green synthesis techniques: top-down and bottom-up. There are various methods for producing NPs and controlling their size, shape, and characteristics. Due to their mild conditions, ease of purification, and high yield, bacteria are ideal for nanoparticle synthesis. These days, bacteria are the most studied microbe, earning the moniker “the factory of nanomaterials.” Bacteria can function as active ingredients in the creation of nanoparticles, biocatalysts, or bioscaffolds (Iqtedar et al. 2019).

Zinc oxide nanoparticles (ZnO NPs), as a type of metal oxide nanoparticle, have attracted considerable interest in biomedical applications due to their broad-spectrum antibacterial properties, favorable toxicity profile toward mammalian cells at effective doses, and diverse modes of action (El-Saadony et al. 2024; Boopathi et al. 2024). The production of reactive oxygen species (ROS), direct physical rupture of the bacterial cell membrane, and the release of Zn^2+^ ions are the main causes of ZnO NPs’ antibacterial impact. This action obstructs essential bacterial metabolic pathways and enzyme functions (Dutta et al. 2012; Abdal Dayem et al. 2017). However, aggregation tendencies, stability issues, and their limited penetration of complex biological structures like biofilms may sometimes impede the stand-alone effectiveness of bare ZnO NPs. To enhance their therapeutic potential, the integration of ZnO NPs into advanced nanocomposite systems has become a significant focus of current research.

Chitosan (CS), a natural polysaccharide known for its biocompatibility, biodegradability, and nontoxic nature, represents an ideal matrix component for nanocomposite formulations (Bamagous et al. 2024). Its polycationic character enables potent electrostatic interactions with the anionic surfaces of bacterial cell membranes and components of the extracellular polymeric substances. This promotes bacterial adhesion, increases membrane permeability, and potentially disrupts the structural integrity of biofilms (Govindasamy et al. 2022; Afsharikhah et al. 2025). Moreover, CS serves as an effective encapsulating or coating agent for both NPs and antibiotics, enhancing their stability, improving solubility, and facilitating targeted delivery (Marín-Silva et al. 2019; Abd-Elraoof et al. 2023). It may be possible to achieve synergistic therapeutic benefits by incorporating traditional antibiotics like amoxicillin (AMX) into these nanocarriers (Enan et al. 2021). The specific target of the beta-lactam antibiotic AMX is bacterial cell wall production (Gibson and Veening 2023). Even though many *P. aeruginosa* strains show resistance to AMX when given alone, its selective distribution within a nanocomposite, when paired with other active drugs, may be able to circumvent current resistance mechanisms and increase overall antimicrobial efficacy. The fundamental idea behind integrating these three different elements—ZnO NPs, CS, and AMX—into a single nanocomposite is to launch a multifaceted assault against *P. aeruginosa*: CS promotes better delivery and membrane disruption, ZnO NPs cause oxidative stress and direct cellular damage, and AMX offers targeted inhibition of cell wall synthesis. This multifaceted approach is designed to overcome bacterial defense strategies and minimize the emergence of further resistance, especially within challenging biofilm formations.

While various commercially available NPs such as Ag, Cu, TiO_2_, and MgO have been explored for antimicrobial applications, the ZnO/CS/AMX nanocomposite was selected for this study due to several critical advantages (Chand and Kushawaha 2024a, b; Govindasamy et al. 2024a, b). Unlike Ag and Cu, which often exhibit significant cytotoxicity toward human cells at therapeutic concentrations, ZnO NPs are recognized for their superior biocompatibility and are Generally Regarded as Safe (GRAS) by the FDA (Brown and Wobst 2021). Furthermore, unlike TiO_2_, which typically requires photoactivation, ZnO maintains high intrinsic bactericidal activity through the continuous generation of ROS (Ivask et al. 2010).

Therefore, the present investigation aimed to synthesize, optimize, and characterize a novel ZnO/chitosan/amoxicillin (ZnO/CS/AMX) nanocomposite. The study also sought to assess its antimicrobial and antibiofilm efficacy against MDR *P. aeruginosa*. Concurrently, the cytotoxicity of the green synthesized ZnO/CS/AMX nanocomposite on mammalian (Vero) cells was evaluated to determine its therapeutic safety profile. The specific aims included the following: (1) fabricating and optimizing the nanocomposite using response surface methodology (RSM) based on the Box-Behnken design (BBD), and conducting physicochemical characterization; (2) determining its antimicrobial potency against *P. aeruginosa* through MICs, MBCs, and agar diffusion assays; (3) examining the ability to inhibit *P. aeruginosa* biofilm formation; and (4) evaluating its in vitro cytotoxicity to establish biocompatibility.

## Materials and methods

### Sample collection

Clinical specimens, including urine, stool, and high vaginal swabs (HVS), were prospectively collected from various private clinics within New Damietta City, Egypt. The collection period spanned 12 months, from January 2024 to December 2024. A total of 44 patient samples were examined for the presence of *P. aeruginosa*. The following inclusion criteria were used to select the samples: patients who were compliant, between the ages of 20 and 50, and had bacterial illnesses. Patients with complex systemic illnesses, immune system abnormalities, physical limitations, or psychiatric issues were excluded from the current study. All patients or their parents or guardians provided informed consent after the study was reviewed and approved by the review boards of the participating institutions. Samples were collected either on the same day or 1 to 2 days after the initial visit. Over the past 3 months, multiple patients had been on antibiotics. The samples were then collected in sterile cups or glass bottles and promptly transported in an icebox to the Microbiology Laboratory at the Faculty of Science, Damietta University in Egypt, for further testing.

### Isolation, culturing, and identification of *Pseudomonas aeruginosa*

In accordance with accepted practices and guidelines, bacterial isolation was conducted in a well-equipped microbiology lab at the Botany and Microbiology Department, Faculty of Science, Damietta University, Egypt. The experimental protocol was approved by the local ethical committee of the Subcommittee on Research Ethics for Basic Sciences at Damietta University, Egypt, in accordance with accepted practices and guidelines. Upon collection, specimens were immediately processed and subcultured onto cetrimide agar plates. They were then incubated aerobically at 37 ℃ for 24 h. Presumptive isolation and identification of *P. aeruginosa* were subsequently performed using standard microbiological methodologies outlined by Zboromyrska and Vila (2016) and Santiago-Rodriguez et al. (2015). The gram-negative, oxidase-positive rods selected on cystine lactose electrolyte deficient (CLED) agar and MacConkey agar were identified at the species level using the Vitek 2 system (BioMerieux, Marcy-l’Étoile, France) (O’Hara 2005). The *P. aeruginosa* clinical isolates utilized in this study are maintained in a −80 °C repository at the Microbiology Laboratory, Botany and Microbiology Department, Faculty of Science, Damietta University.

### Antibiotic sensitivity test

The antibiotic sensitivity test (AST) of the *P. aeruginosa* isolates was evaluated using the disc diffusion technique (CLSI 2018). Fity microliters of each bacterial suspension (0.5 MacFarland standard, 1.5 × 10^8^ CFU/ml) was added to Mueller-Hinton agar (MHA, Oxoid Ltd., England) flasks, which were then placed in sterile Petri dishes. Following solidification, antibiotic discs from different classes were aseptically applied. These included amoxicillin (30 μg/ml), amoxicillin/clavulanate (20/10), ampicillin (30 μg/ml), imipenem (30 μg/ml), cefotaxime (30 μg/ml), ceftazidime (30 μg/ml), chloramphenicol (30 μg/ml), doxycycline (30 μg/ml), levofloxacin (5 μg/ml), nalidixic acid (30 μg/ml), azithromycin (15 μg/ml), meropenem (10 μg/ml), and nitrofurantoin (300 μg/ml). The diameter of the zones of inhibition (ZOI) was measured and recorded in millimeters after a 24-h incubation period at 37 °C.

### Detection of biofilm formation

The crystal violet method was utilized to detect the biofilm development of *P. aeruginosa* isolates in tryptic soy broth (TSB, Oxoid Ltd., England) as described by Kwiecinska-Piróg et al. (2014). Aliquots of a 0.5 MacFarland bacterial suspension (20 μl) were placed into 96-well polystyrene titer plates and mixed with 180 μl of TSB. The cultures were then incubated at 37 °C for 24 h. After incubation, cultures were removed from the wells and plates were allowed to air dry at 37 °C. The plates were then washed with sterile distilled water, and methanol was added to the wells and shaken thoroughly for 20 min at 400 rpm. Once the liquids were discarded from the wells, the plates were again allowed to air dry at 37 °C. The wells were filled with a 0.1% crystal violet solution and then shaken for 10 min at 400 rpm. Following this, a thorough water rinse was performed to remove the crystal violet. The plates were then left to dry for 20 min at 37 °C. Methanol was poured into the wells and shaken for 4 min. The absorbance was measured by spectrophotometry at 570 nm.

### Extracellular biosynthesis of zinc oxide nanoparticles

The isolates of *P. aeruginosa* were inoculated with a 0.5 McFarland standard in sterile nutrient broth flasks. The flasks were then incubated at 37 °C and 150 rpm for 24 h. Centrifugation was used for 20 min at 5000 rpm to collect the bacterial supernatants after the incubation period. A 0.22-μm syringe filter (Millex GV, Millipore) was used for filtering. Separate zinc nitrate solutions (3 mM, ≥ 99.0%, Sigma-Aldrich, USA) were added to the cell-free supernatants. They were then incubated for 24 h at 37 °C and 150 rpm. After the incubation, the reaction mixture was centrifuged for 15 min at 4000 rpm to remove the supernatant. The ZnO NPs were dried for 24 h at 80 °C after being cleaned at least three times with distilled water. The ZnO NP powder was then calcined at 500 °C for 3 h (El-Zahed et al. 2024a).

### Response surface methodology design

The RSM technique based on the BBD with four independent parameters was utilized to investigate and determine the optimal conditions for the production of ZnO NPs (Box and Behnken 1960; Myers et al. 2016). The four key synthesis parameters chosen were as follows: *X*_1_, temperature; *X*_2_, pH; *X*_3_, Na_2_SeO_3_ concentrations; and *X*_4_, the ratio of Na_2_SeO_3_ to cell-free bacterial metabolites. Three levels of each factor were − 1 (low), 0 (middle), and + 1 (high), as determined from initial single-factor trials (Table S1). To minimize the influence of systematic error, the BBD generated a total of 27 experimental runs, which included 6 center points.

In order to model the relationship between the independent factors and the surface Plasmon resonance (SPR) intensity, the experimental data were fitted to a second-order quadratic polynomial equation (Eq. 1):

1 $$Y=\beta_0+{\textstyle\sum_{i-1}^k}\;\beta_i\;X_i+{\textstyle\sum_{i=1}^k}\;\beta_{ii}\;X_i^2+{\textstyle\sum_{i<j}^k}\;X_i\;X_j$$

where *X*_i_ and *X*_j_ are the independent variables, *Y* is the SPR intensity, *k* is the number of independent factors, *β*_0_ is the intercept term, *β*_i_ are the linear coefficients, *β*_ii_ are the quadratic coefficients, and *β*_ij_ are the interaction coefficients.

The significance of the quadratic model, the individual elements, and their interactions were assessed using analysis of variance (ANOVA) at a 95% confidence level (*p* < 0.05). The coefficient of determination and the lack of fit test were used to evaluate the model’s quality. Statistical analysis was conducted using Minitab Statistical Software (version 22; Minitab, LLC, State College, PA), which included ANOVA, model fitting, and creation of the RSM plots (Montgomery 2017).

### Synthesis of zinc oxide/chitosan/amoxicillin nanocomposite

**Fig. 1:**
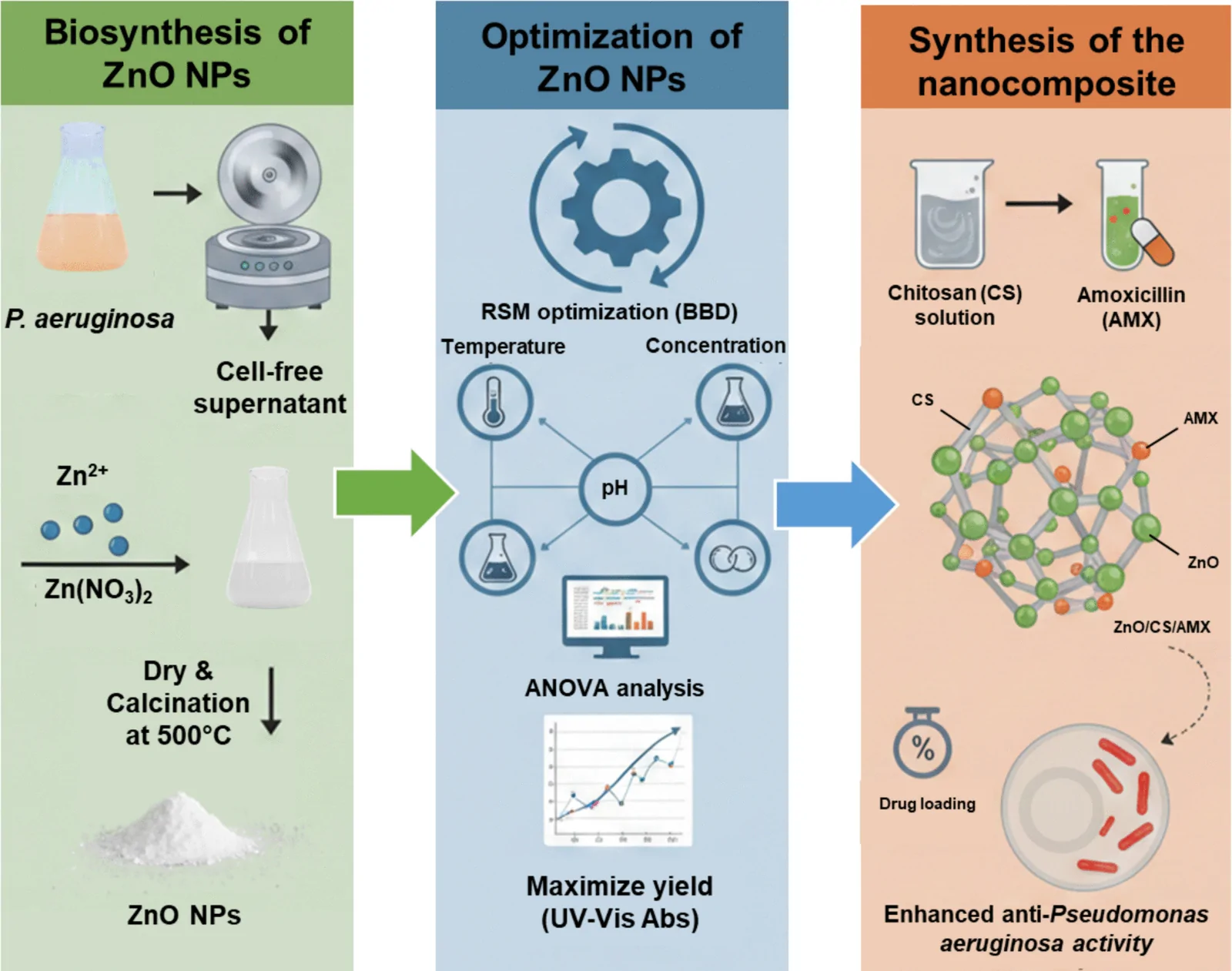
Sequential steps for the green synthesis of amoxicillin-loaded ZnO/chitosan nanocomposite (ZnO/CS/AMX)

A 0.1% CS solution with a molecular weight of 50–190 kDa, (Sigma-Aldrich, USA) was prepared at room temperature using 1% acetic acid. The solution was stirred overnight until fully dissolved. ZnO NPs were then added to the CS solution and stirred overnight as well (Al-Naamani et al. 2017). Next, an equivalent amount of the antibiotic AMX (Mylan Pharmaceuticals Ltd., Ireland) was introduced to the zinc oxide/chitosan solution at room temperature. The mixture was agitated at 5000 rpm throughout the night. Centrifuging the ZnO/CS/AMX nanocomposite resulted in three rounds of distilled water washing for the residue. The final step was to dry the product for 24 h at 50 °C (Elshikiby et al. 2025). The drug loading of the ZnO/CS/AMX was determined by dividing the weight of AMP loaded into the ZnO/CS/AMX by the overall weight of ZnO/CS/AMX. A schematic diagram illustrating the green synthesis of the ZnO/CS/AMX nanocomposite is displayed in Fig. 1.

### Characterization of zinc oxide/chitosan/amoxicillin nanocomposite

The ZnO/CS/AMX nanocomposite was analyzed using Fourier transform infrared spectroscopy (FTIR, FT/IR-4100typeA) and a double beam UV–Vis spectrophotometer V-630 (JASCO, UK). In addition, a zeta potential analyzer (Malvern Zetasizer Nano-ZS90, Malvern, UK), a transmission electron microscope (TEM) (200 kV, JEOL JEM-2100, Japan), and an X-ray diffractometer (model LabX XRD-6000, Shimadzu, Japan) were utilized to investigate the properties of ZnO/CS/AMX.

### Anti-*Pseudomonas aeruginosa* activity using agar well diffusion method

Using the agar well diffusion method, we examined the antibacterial activity of ZnO/CS/AMX against various *P. aeruginosa* isolates and *P. aeruginosa* ATCC 27853 comparing it to ZnO NPs (CLSI 2017). MHA plates were inoculated with a single strain of bacteria. Dimethyl sulfoxide (DMSO) was used to create a concentration of 50 μg/ml for each antibacterial agent tested. This solution was then added to wells on MHA plates and incubated for 48 h at 37°C. Imipenem was utilized as the positive control, while DMSO served as the negative control. The resulting zones of inhibition (ZOI) were measured in millimeters (mm). To determine the fold increase in area for ZnO NPs combined with CS and AMX, the formula (*B*^2^*–A*^2^)/*A*^2^ was used. Here *A* represents the ZOI of ZnO NPs alone, and *B* represents the ZOI of ZnO NPs mixed with CS and AMX (Baka et al. 2024).

### Minimum inhibitory concentration

Mueller-Hinton broth (MHB) medium was utilized to determine the minimum inhibitory concentration (MIC) values for ZnO NPs and ZnO/CS/AMX against the tested bacterial strains through the broth dilution method (CLSI 2000). Initially, 0.5 MacFarland of each bacterial strain was introduced into MHB, along with various concentrations of ZnO NPs and ZnO/CS/AMX (0–150 μg/ml). The mixture was then placed in an incubator at 37 °C and 150 rpm. After a 24-h incubation period, the MIC values for ZnO NPs and ZnO/CS/AMX were determined using spectrophotometry at 600 nm.

### Minimum bactericidal concentration

Fifty-microliter aliquots from each tube that showed no discernible bacterial growth (MIC) were seeded onto MHA plates using the pour plate method. The plates were then incubated at 37 °C for 24 h. Following the evaluation of bacterial colony growth, the total colony-forming units per milliliter (CFU/ml) were counted in order to calculate minimum bactericidal concentration (MBC) values (Owuama 2017).

### Determination of fractional inhibitory concentration index

To quantitatively evaluate the interaction between the components within the ZnO/CS/AMX nanocomposite, the fractional inhibitory concentration index (FICI) was calculated based on the MIC values of the individual agents and the formulation. The FICI was determined using the following formula:

$$FICI=\frac{MIC_{Nanocomposite}}{MIC_{ZnO}}+\frac{MIC_{Nanocomposite}}{MIC_{AMX}}$$

The results were interpreted as follows: synergy (FICI ≤ 0.5), additivity (0.5 < FICI ≤ 1.0), indifference (1.0 < FICI ≤ 4.0), and antagonism (FICI > 4.0).

### Ultrastructural study using TEM

Using *P. aeruginosa* ATCC 27853 as a standard bacterial model, the mechanism of action of ZnO NPs and ZnO/CS/AMX was examined in order to determine their antibacterial capabilities. To ensure the reliability of the qualitative data, three independent experiments were performed for each treatment group (control, ZnO NPs, and ZnO/CS/AMX). MHB flasks were inoculated with a 0.5 MacFarland suspension of *P. aeruginosa* following treatment with MBC of each nanomaterial. The flasks were then subsequently incubated for 24 h at 37 °C and 150 rpm. After adding ZnO NPs or ZnO/CS/AMX, the bacteria were allowed to multiply for 2 h. The cells were then produced through centrifugation at 8000 rpm for 20 min in an aseptic setting. As a standard procedure, the cells were washed with sterile distilled water. Bacterial cells were then fixed for 20 min at pH 7 using 2.5% glutaraldehyde in 0.1 M cacodylate buffer before being extracted. Samples were double-stained and viewed using a TEM on carbon-coated copper grids (Type G 200, USA) after the preserved cells had been dried with a series of ethanol (Egerton 2016). Grids were systematically scanned and a minimum of 50 fields containing at least 200 individual cells were examined per sample using TEM. Micrographs were selected based on their representativeness of the dominant cellular phenotypes observed across the entire specimen.

### Biofilm formation inhibition assay

The antibiofilm ability of ZnO NPs and ZnO/CS/AMX was assessed against various strains of *P. aeruginosa* using the crystal violet staining method to quantify the inhibition of biofilm formation. Specifically, 100 μl of 1% w/v glucose-TSB was added to wells of 96-well polystyrene titer plates, followed by 10-μl aliquots of bacterial suspension (0.5 MacFarland) as the inoculant. After independently adding 50, 100, and 150 µg/ml of ZnO NPs or ZnO/CS/AMX to the wells, they were incubated for 24 h at 37°C. Controls were also prepared, with media containing a bacterial suspension in the positive control well, and sterile medium only in the negative control well. Following incubation, the cultures were removed and the wells were cleaned with sterile distilled water. The residual biofilm was stained with crystal violet (0.25%) and ethanol (95%). The absorbance of the cells-dye combination was then measured at 570 nm using spectrophotometry. The biofilm inhibitory percentage was calculated using the formula: antagonistic effectiveness = (*A*_1_*–A*_2_)/*A*_1_ × 100, where *A*_1_ and *A*_2_ are the absorbance values of the treated and untreated samples, respectively (Achudhan et al. 2020).

### Cytotoxicity activity

ZnO NPs and ZnO/CS/AMX were tested for their cytotoxic effects on Vero cells (ATCC, Rockville, MD). The Vero cells were cultured in Dulbecco’s Modified Eagle’s medium (DMEM) supplemented with 1% L-glutamine, 10% heat-inactivated fetal bovine serum, and 50 μg/ml gentamicin. In the exponential growth phase, 1 × 10^4^ cells per well in 100 μl of growth media were seeded into 96-well tissue culture plates using a multichannel pipette. After that, the cells were left to adhere for a full day. ZnO NPs or ZnO/CS/AMX were then added to the respective wells to reach final concentrations ranging from 0 to 500 μg/ml. Doxorubicin (DOX) was used. Following a 24-h incubation period at 37 °C in an incubator with 5% CO_2_, the number of live cells was determined using the MTT assay, as described by Mosmann (1983). Survival curves for each cell line were created after treatment with ZnO NPs or ZnO/CS/AMX by plotting the relationship between the concentration of these substances and the remaining cells.

### Statistical analysis

The data was statistically evaluated using SPSS version 18 software. Each experimental value was presented as the mean ± standard deviation (SD) in a one-way analysis of variance (ANOVA). A significance level of less than 0.05 was set, and either the least significant difference (LSD) test or Duncan’s multiple range test was used for intergroup comparisons. Each experiment was run three times.

## Results

### Isolation and phenotypic characterization of *P. aeruginosa* isolates

On cetrimide agar, 11 *P. aeruginosa* isolates appeared as yellow-green to blue colonies after a 24-h incubation period. These bacteria were non-spore forming and negative for lactose fermentation, but positive for citrate and glucose fermentation. Additionally, MacConkey and CLED agar plates were used for their culturing. MacConkey agar plates were utilized to distinguish these bacteria, which appeared as flat, round, and colorless colonies. This medium’s components, such as crystal violet and bile salts, are believed to have a selective impact. They inhibit the growth of most gram-positive bacteria while promoting the growth of gram-negative ones. Additionally, colonies of *P. aeruginosa* that were grown on CLED agar plates appeared colorless, pale green, or blue-green (Atlas and Snyder 2013). Species-level identification using Vitek 2 revealed the presence of 11 isolates of *P. aeruginosa* labeled as YMA1, YMB4, YMB5, YMC18, YMC21, YMD2, YMD5, YMD6, YME1, YME2, and YMF7 (Table S2). Each *P. aeruginosa* isolate was tested for ZnO NPs synthesis.

### AST pattern for *P. aeruginosa* isolates

The patterns of antibiotic sensitivity and resistance in *P. aeruginosa* isolates are shown in Table 1 and Fig. S1. According to CLSI standards, the percentages of susceptibility and resistance were determined. For instance, imipenem susceptibility is indicated by an inhibition zone of ≥ 16 mm. The reference strain ATCC 27853 exhibited high susceptibility (35 ± 0.03 mm), but several clinical isolates displayed decreased susceptibility or resistance emerging. For example, YMB5 (21 ± 0.14 mm) remains within the susceptible range. It is important to note that all isolates were completely resistant to AMX alone, underscoring the inherent challenges in treating these clinical strains. The results indicate that the *P. aeruginosa* YMB5 exhibits greater resistance to the major tested antibiotics compared to the other strains, with *P. aeruginosa* YMC18, *P. aeruginosa* YMC21, and *P. aeruginosa* YMD6 following in resistance level. In addition, all isolates of *P. aeruginosa* show 100% resistance to ampicillin. On the other hand, more than 90% of the isolates were susceptible to azithromycin, chloramphenicol, levofloxacin, and meropenem. In particular, only one isolate (*P. aeruginosa* YMB5) exhibited resistance to meropenem, azithromycin, and chloramphenicol; the other ten were susceptible. In a similar vein, only *P. aeruginosa* YMB5 was resistant to levofloxacin, while the other ten isolates were susceptible. Amoxicillin/clavulanate was effective against 5 *P. aeruginosa* isolates (YMA1, YMB4, YMD2, YME1, and YMF7), with a sensitivity rate of about 45.5% and a resistance rate of 54.5%. Only 4 *P. aeruginosa* isolates (YMA1, YMB4, YME2, and YMF7) were sensitive to cefotaxime, indicating a sensitivity of roughly 36.4% and resistance of 63.6%. With 5 susceptible *P. aeruginosa* isolates (YMA1, YMB4, YMD2, YME1, and YMF7) and 6 resistant isolates, ceftazidime exhibited a profile comparable to that of amoxicillin/clavulanate. Only *P. aeruginosa* YMB5 and *P. aeruginosa* YMC18 were resistant to nalidixic acid, which was effective against the other isolates with a sensitivity of about 81.8%.

**Table 1:** Patterns of antibiotic resistance and sensitivity in *P. aeruginosa* isolates

Antibiotic	Concentration (µg/ml)	Antibiotics susceptibility^*^										
		YMA1	YMB4	YMB5	YMC18	YMC21	YMD2	YMD5	YMD6	YME1	YME2	YMF7
Amoxicillin	30	R	R	R	R	R	R	R	R	R	R	R
Amoxicillin/clavulanate	20/10	S	S	R	R	R	S	R	R	S	R	S
Ampicillin	30	R	R	R	R	R	R	R	R	R	R	R
Azithromycin	15	S	S	R	S	S	S	S	S	S	S	S
Cefotaxime	30	S	S	R	R	R	R	R	R	R	S	S
Ceftazidime	30	S	S	R	R	R	S	R	R	S	R	S
Chloramphenicol	30	S	S	R	S	S	S	S	S	S	S	S
Doxycycline	30	S	R	R	R	R	R	R	R	R	R	S
Imipenem	30	S	R	S	R	R	S	S	R	R	R	R
Levofloxacin	5	S	S	R	S	S	S	S	S	S	S	S
Meropenem	10	S	S	R	S	S	S	S	S	S	S	S
Nalidixic acid	30	S	S	R	R	S	S	S	S	S	S	S
Nitrofurantoin	300	S	R	R	R	R	R	R	R	R	R	R

^*^S, susceptible; *R*, resistant

### Biofilm formation ability of *P. aeruginosa* isolates

**Fig. 2:**
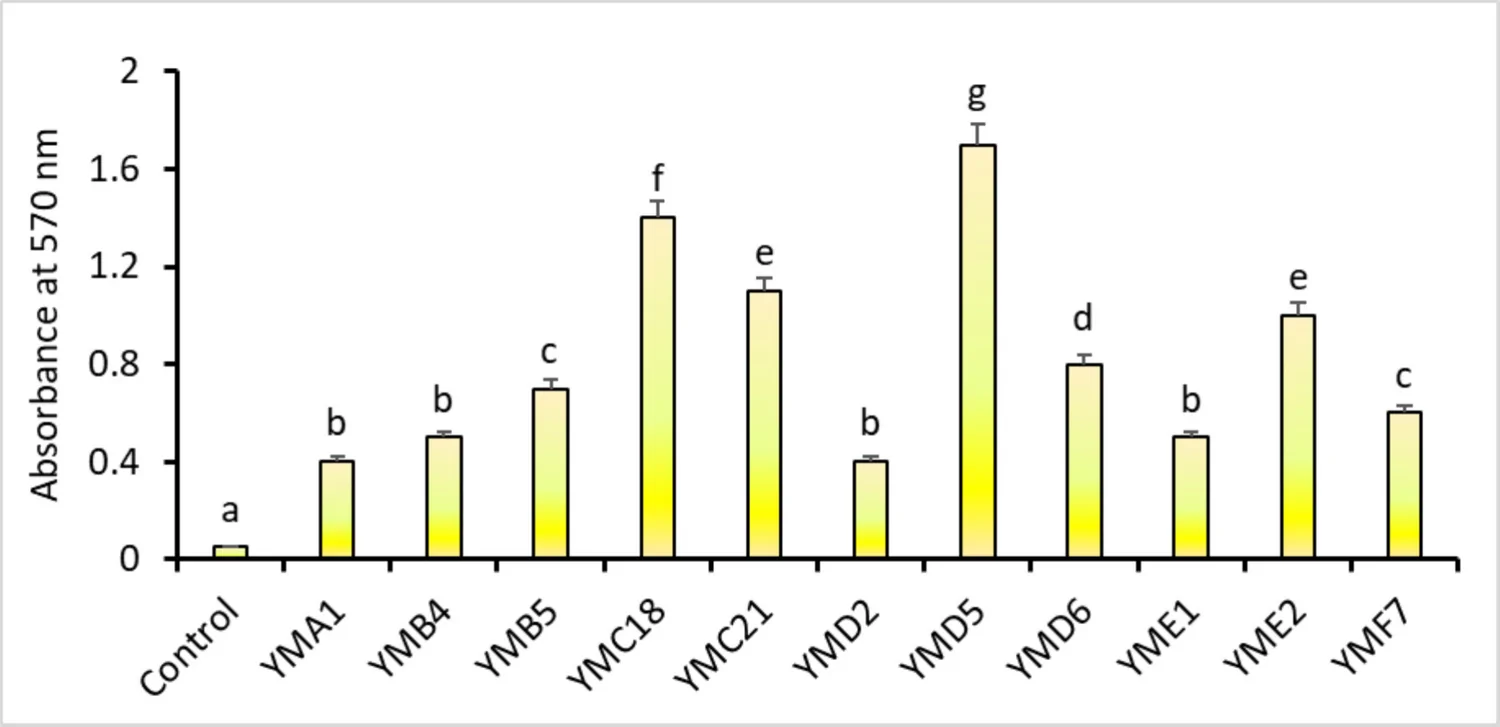
Quantitative assessment of *P. aeruginosa* biofilm formation. Bars represent the mean, and error bars indicate the SD of the mean. Different letters above the error bars indicate a statistically significant difference between sample groups (*p* < 0.05) based on LSD post-hoc analysis

The ability of different *P. aeruginosa* isolates to form biofilms was quantitatively assessed using the crystal violet method (Fig. 2). *P. aeruginosa* YMD5 showed the strongest biofilm-promoting effect, followed by *P. aeruginosa* YMC18, *P. aeruginosa* YMC21, and *P. aeruginosa* YME2. Statistical analysis using LSD post-hoc test confirmed that *P. aeruginosa* YMD5 was significantly different from all other isolates, highlighting its potent biofilm-enhancing capacity. While *P. aeruginosa* YMC21 and *P. aeruginosa* YME2 showed statistically indistinguishable biofilm-promoting effects from each other, *P. aeruginosa* YMA1, *P. aeruginosa* YMB4, YMD2, and *P. aeruginosa* YME1 consistently exhibited lower biofilm formation compared to the other strains.

### Synthesis of ZnO NPs by *P. aeruginosa* isolates

**Fig. 3:**
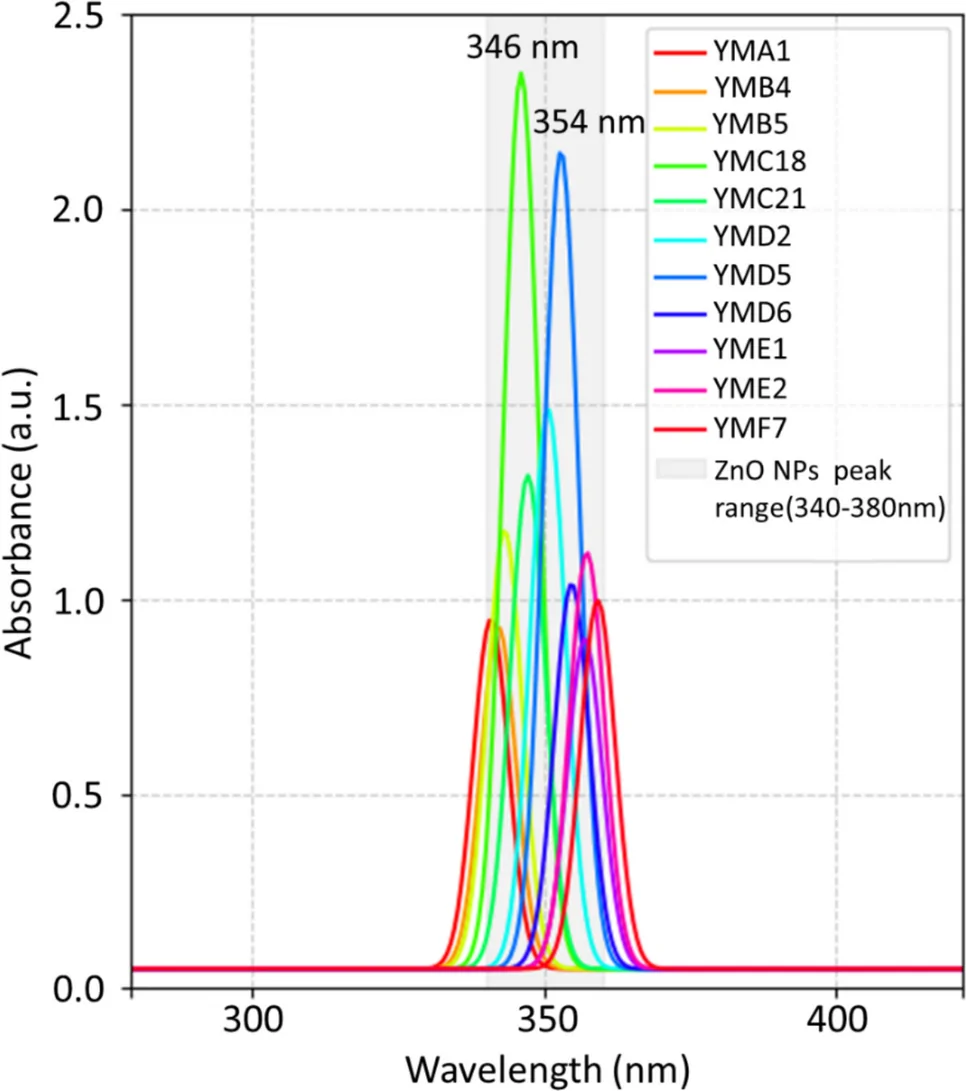
UV–Vis spectroscopy during the formation of ZnO NPs using *P. aeruginosa* isolates (YMA1-YMF7). *P. aeruginosa* YMC18 and *P. aeruginosa* YMD5 revealed the highest ZnO NP production rate at wavelengths of 346 nm and 354 nm, respectively

The color of the reaction mixture changed from pale yellow at the beginning of the experiment to a white colloidal suspension at the end of the incubation period while evaluating the formation of ZnO NPs by the *P. aeruginosa* isolates (Fig. 3). ZnO NP synthesis was verified by UV–Vis spectrometric measurement, where absorbance peaks ranging from 340 to 380 nm were observed during the production of ZnO NPs using the cell-free metabolites from various *P. aeruginosa* isolates. Quantitative analysis of the SPR peak intensities revealed significant differences in ZnO NP production rates among the *P. aeruginosa* isolates. Specifically, *P. aeruginosa* YMC18 and *P. aeruginosa* YMD5 exhibited the highest production rates compared to the other bacterial isolates. In contrast, *P. aeruginosa* isolates such as YMA1, YMB4, YMD2, and YME1 showed comparatively lower SPR intensities, indicating less efficient biosynthesis of ZnO NPs.

### Optimization of ZnO NPs production using RSM

The relationship between the SPR intensity and the four independent variables including *X*_1_ (temperature), *X*_2_ (pH), *X*_3_ (concentration of zinc nitrate), and *X*_4_ (mixing ratio between zinc nitrate and cell-free bacterial metabolite) is described by the following equation (Eq. 2):

2 $$\begin{aligned}Y&=5.9823+0.0977\;X_1+0.1026\;X_2-0.4297\;X_3-0.0129\;X_4-0.0017\;X_1^2\\&-0.1556\;X_2^2\;-0.0006\;X_3^2\;-0.0082\;X_4^2\;+0.00014\;X_1\;X_2+0.00032\;X_1\;X_3\\&+0.0051\;X_1\;X_4+0.0694\;X_2\;X_3-0.001\;X_2\;X_4\end{aligned}$$

The concentration of zinc nitrate had the most pronounced linear influence. The significant negative sign indicates that increasing the Zn precursor content has a detrimental effect on the SPR intensity. Additionally, the pH showed a significant impact with a positive linear coefficient of + 0.1026, suggesting a positive linear effect on the production of ZnO NPs. The dominant negative quadratic coefficient of pH significantly moderates this linear relationship, indicating a significant nonlinear relationship where pH needs to be carefully adjusted around the center level. In contrast, the linear effects of temperature and the ratio of mixing between the cell-free bacterial metabolite and zinc nitrate had a lesser impact. The pH and concentration interactions were the most significant among the interaction terms, indicating that the synergistic effect between the medium’s pH and the ZnO precursor concentration is crucial for optimizing the production of ZnO NPs.

ANOVA was used to thoroughly assess the validity and statistical significance of the model, as illustrated in Table S3. A *p*-value of 0.0384 (*p* < 0.05) and an *F*-value of 3.01 indicated that the overall model was statistically significant. The non-significant lack of fit *p*-value of 0.9997 (*p* > 0.10) suggests that the model effectively describes the experimental data and that the residual error is simply random noise, further bolstering the model’s fitting capability. The coefficient of determination was 0.7760, indicating that the parameters being investigated could account for 77.60% of the variation in the SPR intensity. The precision value of 5.321 was significantly higher than the essential threshold of 4.0, confirming an adequate signal-to-noise ratio needed for navigating the design space and further supporting the model’s reliability. Ultimately, the strong significance of the quadratic terms (*p*-value = 0.0006) supported the original decision to use a second-order model. This demonstrates that the relationship between SPR intensity and synthesis parameters is nonlinear and requires optimization to achieve the maximum ideal value.

The internally studentized residuals lie along the 45° line, as depicted in Fig. S2A, confirming the fundamental assumption of normally distributed errors and ensuring the reliability of the *p*-values derived from the ANOVA. Furthermore, Fig. S2B illustrates a random distribution of residuals centered around the zero line across the entire range of responses, indicating that the model is unaffected by the anticipated response magnitude and confirming constant variance (homoscedasticity).

**Fig. 4:**
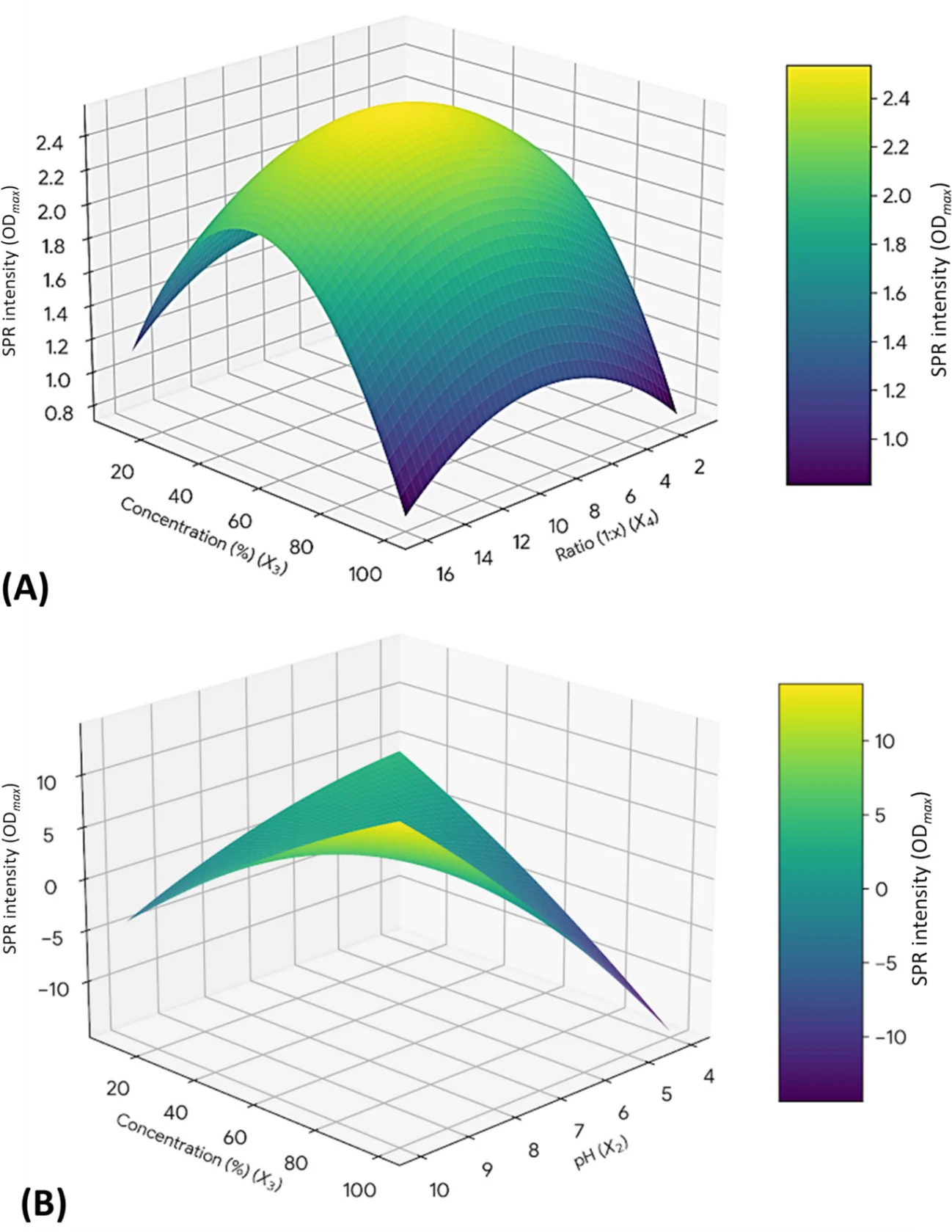
3D response surface plots illustrating the combined effects of synthesis parameters on the SPR intensity (OD_max_) (ZnO NPs yield). **A** Response surface showing the interaction effect between the concentration of zinc nitrate and the mixing ratio between zinc nitrate and cell-free bacterial metabolites, with the other factors held constant at their center points. **B** Response surface showing the interaction effect between the concentration of zinc nitrate and pH, with the other factors held constant at their center points

Figure 4 displays a 3D RSM plot illustrating the combined impact of zinc nitrate concentration and the ratio of mixing between zinc nitrate and cell-free bacterial metabolite on the SPR intensity. The plot clearly shows a significant nonlinear interaction between these two parameters. The combination of a low zinc nitrate concentration and a low mixing ratio between the zinc nitrate and cell-free bacterial metabolite results in the highest projected SPR intensity. The SPR intensity (and therefore ZnO NPs yield) is extremely sensitive to increasing precursor concentration, as shown by the noticeable gradient, particularly along the concentration axis. Regardless of the ratio, the response surface quickly decreases toward low-intensity areas as the concentration rises toward the upper limit of the design space. This critical sensitivity indicates that conditions of low initial precursor concentration are optimal for maintaining the required colloidal stability and particle morphology, as measured by SPR intensity. This is likely due to reduced aggregation and controlled growth kinetics. The maximum response observed empirically was found to be near the central conditions through numerical analysis using the fitted quadratic model. The final recommended optimal synthesis parameters were determined to be 30 °C (temperature), 7.0 (pH), 30 mM (zinc concentration), and 1:4 (mixing ratio between zinc nitrate and cell-free bacterial metabolite) due to the stability of the center point and minimal degradation in SPR intensity compared to extreme boundary conditions. This condition, centered around a neutral pH value, offers the ideal compromise between optimizing SPR intensity and ensuring industrial scalability of the process.

### Characterization of ZnO NPs and ZnO/CS/AMX

**Fig. 5:**
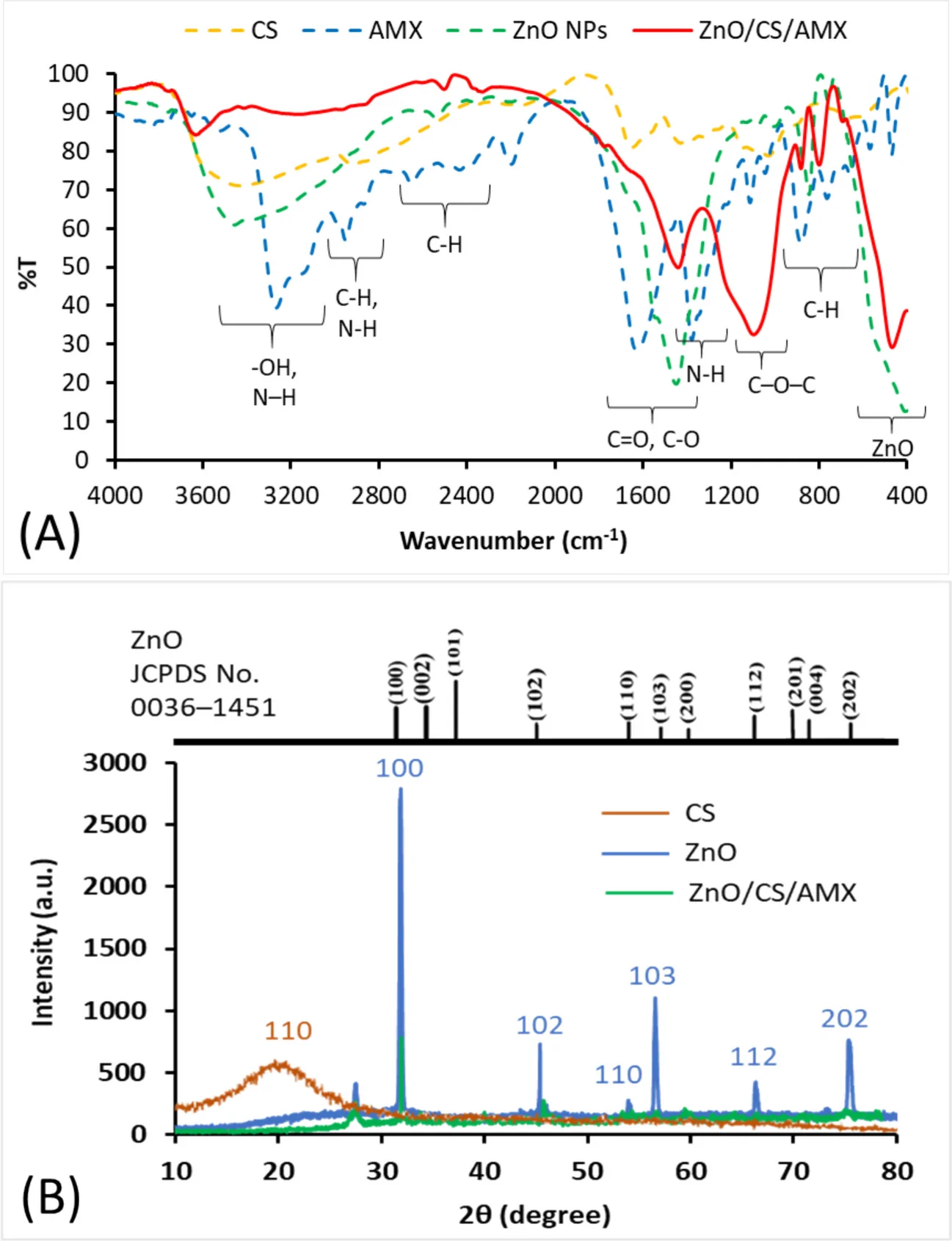
**A** FTIR spectra of CS, AMX, ZnO NPs, and ZnO/CS/AMX. **B** XRD patterns of ZnO NPs and ZnO/CS/AMX

Biosynthesized ZnO NPs produced by *P. aeruginosa* YMC18 and ZnO/CS/AMX were characterized using FTIR, XRD, TEM, and zeta potential analyses. The FTIR spectrum of CS shows distinctive broad absorption bands of -OH and NH_2_ groups, which are responsible for the noticeable broad band seen at about 3400 cm^−1^ (Fig. 5A). Bands in the 1650 cm^−1^ and 1590 cm^−1^ regions are linked to C = O stretching (amide I) and N–H bending (amide II) from residual N-acetyl groups, respectively, coupled with C-O stretching at approximately 1050 cm^−1^. The peaks at about 2870 cm^−1^ correspond to C-H stretching. The stretching vibrations of -OH groups in AMX are represented by a broad band at approximately 3400–3200 cm^−1^. C-H stretching is responsible for peaks in the 2900–2800 cm^−1^ range. The beta-lactam C = O stretching is characterized by a notable strong absorption peak at 1770 cm^−1^, while the amide C = O stretching (amide I) of the side chain is represented by another strong peak at 1680 cm^−1^. The fingerprint area usually contains additional peaks associated with aromatic rings and other functional groups. A prominent and wide absorption band that is usually seen below 600 cm^−1^, more precisely about 450 cm^−1^, characterizes the spectra of ZnO NPs. Surface adsorbed water molecules are typically responsible for the broad band at 3400 cm^−1^ and smaller peaks in the 1630 cm^−1^ region for ZnO NPs. The FTIR spectrum of ZnO/CS/AMX nanocomposite provides crucial evidence of the successful integration and interactions of the three components. There is still a noticeable Zn–O stretching vibration at around 450 cm^−1^, indicating the presence of ZnO NPs in the composite structure. The characteristic regions of CS and AMX exhibit significant shifts and changes. The broad -OH and NH_2_ stretching bands of CS (around 3400 cm^−1^) shift and widen, implying hydrogen bonding and electrostatic interactions between CS, AMX, and ZnO NPs (Elshikiby et al. 2025). The amide C = O stretching band (around 1680 cm^−1^) is also affected, and the distinct beta-lactam C = O stretching of AMX at about 1770 cm^−1^ is weakened and slightly shifted.

The XRD pattern of the ZnO NPs clearly exhibits several sharp and intense diffraction peaks, which are characteristic of a highly crystalline nanomaterial (Fig. 5B). The crystal planes (100), (101), (102), (103), (110), (112), and (202) are represented by the significant diffraction peaks at 2*θ* values. The conventional diffraction pattern of hexagonal wurtzite ZnO (JCPDS card No. 0036-1451) matches well with these observed peak positions and relative intensities. Using the Scherrer equation, the average crystallite size of the ZnO NPs on the (101) plane was calculated, resulting in a quantitative crystalline size of 59 nm. The ZnO peaks are still visible in the ZnO/CS/AMX XRD pattern, but their reduced intensity and the presence of a wider, less distinct profile suggest that the ZnO NPs are either highly dispersed or effectively encapsulated within the amorphous CS and AMX matrix. The amorphous structure of CS and the organic AMX drug, which lack distinct diffraction peaks, are reflected in the broad humps and diffuse appearance of the ZnO/CS/AMX composite spectrum. Instead of just physical mixing where distinct ZnO peaks would remain with high intensity, the successful incorporation of ZnO NPs into the polymeric/drug matrix is confirmed by this decrease in crystallinity and the masking effect. On the other hand, the CS pattern appears to show a broad, less intense peak with the (110) plane at about 2*θ*≈20–22°, which is typically associated with the semi-crystalline structure of CS.

Spectrophotometric analysis was used to determine the maximum drug loading of AMX in the ZnO/CS/AMX. By plotting known concentrations against their corresponding absorbance values, a standard curve of pure AMX in distilled water was created (Fig. S3). The quantity of AMX present in the nanocomposite samples was determined using the formula derived from the standard curve. ZnO/CS/AMX exhibited a drug loading of 55.7%.

**Fig. 6:**
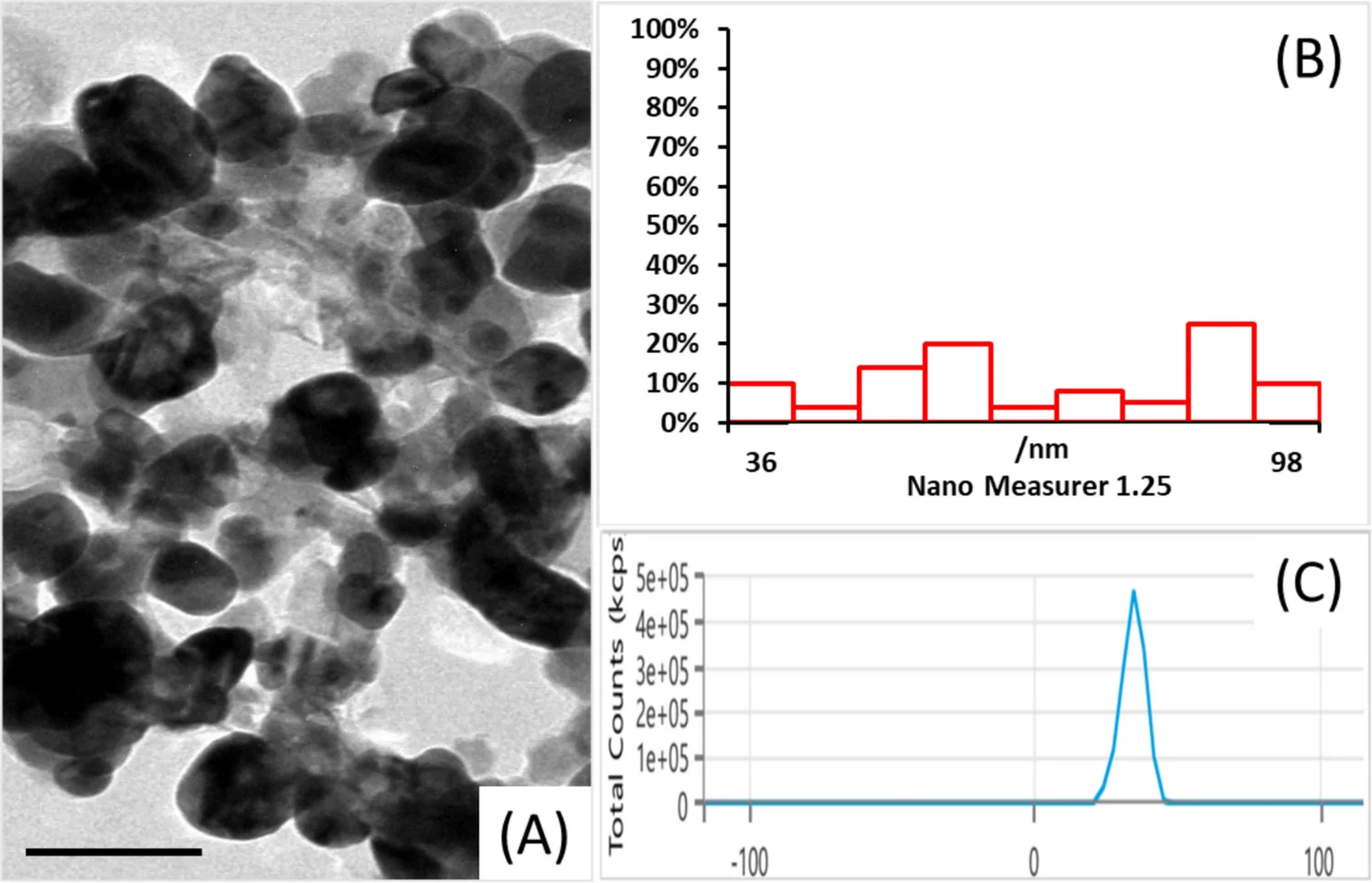
**A** TEM micrographs of ZnO/CS/AMX; bar scale = 100 nm. **B** Nanogravimetric image showing the particle size distribution. **C** Zeta potential of ZnO/CS/AMX

The TEM micrograph of the ZnO/CS/AMX shows that the particles are mostly spherical to quasi-spherical in shape; however, some irregular shapes may also be present (Fig. 6A). The particle size distribution profile is displayed as a nanogravimetric image in Fig. 6B. The particles range approximately 36 to 98 nm, indicating a relatively broad size distribution. There are prominent peaks around 50–60 nm and another broader peak near 80–90 nm, suggesting a somewhat multimodal distribution. The zeta potential of the ZnO/CS/AMX nanocomposite is shown in Fig. 6C. The nanocomposite particles exhibit a net positive surface charge, indicated by the clear, sharp peak at a positive zeta potential value of + 35 ± 2.3 mV.

### Anti-*Pseudomonas aeruginosa* activity

The antibacterial efficacy of ZnO NPs, ZnO/CS/AMX, and imipenem was rigorously evaluated against various *P. aeruginosa* clinical isolates as well as *P. aeruginosa* ATCC 27853 using the agar well diffusion method, MIC, and MBC tests. According to the agar well diffusion investigations, imipenem, a cornerstone carbapenem frequently used against *P. aeruginosa*, showed varying degrees of inhibition. The reference strain *P. aeruginosa* ATCC 27853 exhibited the highest susceptibility response to all treatments (Fig. S4). Imipenem created a large inhibition zone of 35 ± 0.03 mm, significantly greater than most clinical isolates, confirming its susceptible phenotype. Similarly, the ZnO NPs and the ZnO/CS/AMX nanocomposite also displayed promising activity against *P. aeruginosa* ATCC 27853, with zones of 25 ± 0.06 mm and 35 ± 0.03 mm, respectively. Remarkably, the nanocomposite’s activity against *P. aeruginosa* ATCC 27853 was statistically indistinguishable from imipenem, highlighting its potent antimicrobial capacity against a fully susceptible strain. Isolates such as YMA1 (29 ± 0.03 mm), YMB5 (21 ± 0.06 mm), YMC21 (21 ± 0.14 mm), YMD2 (26 ± 0.03 mm), YME1 (25 ± 0.03 mm), YME2 (22 ± 0.06 mm), and YMF7 (19 ± 0.21 mm) displayed clear susceptibility to imipenem (zone ≥ 16 mm according to the CLSI standards), though their response was significantly lower than the reference strain ATCC 27853.

On the other hand, ZnO NPs exhibited the lowest antibacterial activity against all isolates, producing inhibition zones ranging from 12 ± 0.25 mm (YMA1) to 14 ± 0.18 mm (YMD2). Crucially, the ZnO/CS/AMX consistently demonstrated significantly enhanced antibacterial activity across all isolates, often surpassing the activity of ZnO NPs alone and, remarkably, overcoming the inherent resistance of the isolates to both CS and AMX individually. For YMA1, the ZnO/CS/AMX exhibited activity (28 ± 0.03 mm) comparable to imipenem (29 ± 0.03 mm). In the case of isolates YMD2 and YME1, ZnO/CS/AMX also showed strong activity (26 ± 0.03 mm and 24 ± 0.06 mm, respectively) similar to imipenem. However, for isolates like YMB4, YMB5, YMC18, YMC21, YMD5, YMD6, YME2, and YMF7, ZnO/CS/AMX demonstrated slightly lower efficacy compared to imipenem.

**Fig. 7:**
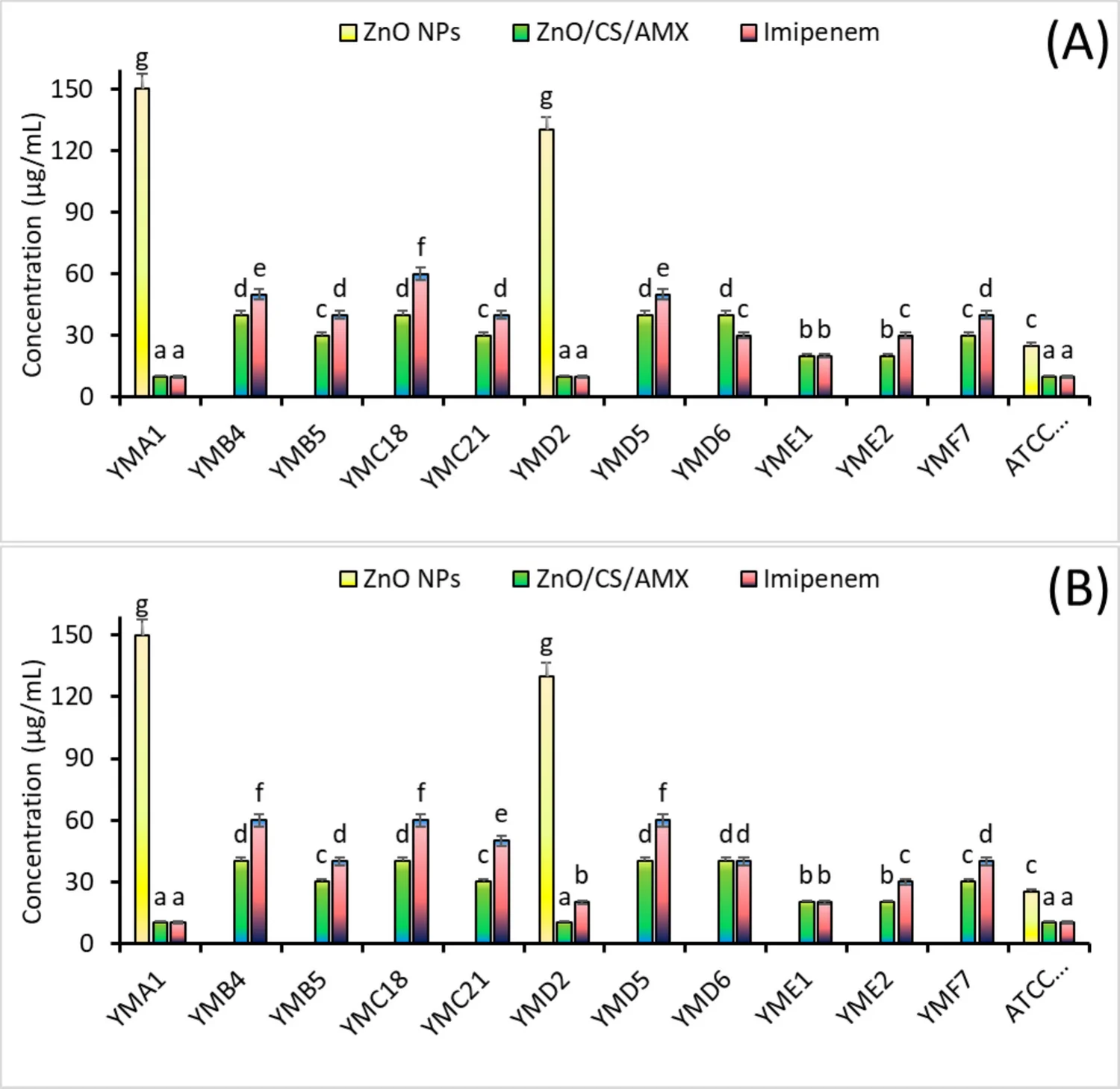
**A** Minimum inhibition concentration of ZnO NPs, ZnO/CS/AMX, and against the tested bacteria. **B** Minimum bactericidal concentration of ZnO NPs, ZnO/CS/AMX, and against the tested bacteria

The antibacterial efficacy of ZnO NPs, ZnO/CS/AMX nanocomposite, and imipenem against various *P. aeruginosa* isolates and the reference strain ATCC 27853 was further determined by assessing their MIC and MBC (Fig. 7). The MIC values for the tested strains varied significantly among the *P. aeruginosa* isolates (Fig. 7A). For imipenem, the reference strain ATCC 27853 exhibited a very low MIC of 10 µg/ml, indicating high susceptibility. In contrast, clinical isolates showed varying responses with different values of MICs. While *P. aeruginosa* YMD6 and YME2 had relatively higher MICs of 30 µg/ml, *P. aeruginosa* YMC18 displayed a significantly higher MIC of 60 µg/ml, suggesting a substantial level of resistance or reduced susceptibility to imipenem compared to *P. aeruginosa* ATCC 27853. For ZnO NPs, MIC against the *P. aeruginosa* ATCC 27853 was 25 µg/ml. However, it is important to note that against *P. aeruginosa* YMA1 and YMD2, ZnO NPs exhibited significantly higher MICs of 150 µg/ml and 130 µg/ml, respectively. This suggests a relatively lower inhibitory effect compared to other treatments. Other *P. aeruginosa* isolates were completely resistant to ZnO NPs. In contrast, the ZnO/CS/AMX nanocomposite consistently showed significantly lower MIC values for almost all tested isolates, particularly when compared to ZnO NPs alone. It often rivaled or surpassed the efficacy of imipenem. The nanocomposite significantly reduced the MIC against *P. aeruginosa* YMA1 to 10 µg/ml compared to 150 µg/ml for ZnO NPs alone, demonstrating a strong synergistic effect. Against *P. aeruginosa* ATCC 27853, the nanocomposite achieved an MIC of 10 µg/ml, which was statistically similar to imipenem. This suggests that the combined action of ZnO NPs, CS, and AMX, facilitated by the nanocomposite structure, effectively enhances the inhibitory action. The MBC values of ZnO/CS/AMX generally followed a similar trend to the MICs, often being slightly higher than their corresponding MICs, as expected for bactericidal agents (Fig. 7B). While ZnO NPs alone showed variable MBCs, with YMA1 and YMD2 requiring significantly higher concentrations (150 µg/ml and 130 µg/ml, respectively) for a bactericidal effect. ZnO/CS/AMX and imipenem showed the lowest MBCs of 10 µg/ml against *P. aeruginosa* ATCC 27853, while other isolates such as *P. aeruginosa* YMB4, YMC18, and YMD5 had significantly higher MBCs (60 µg/ml), reinforcing their reduced susceptibility.

The latest results indicate that the ZnO/CS/AMX combination achieved MICs as low as 10 µg/ml. For example, this was observed in *P. aeruginosa* ATCC 27853, YMA1, and YMD2. Additionally, the MBCs were often equivalent to or slightly higher than these MICs. This represents a significant reduction compared to the effects of the individual components. The synergistic enhancement, where the composite’s MICs are significantly lower than the MICs of ZnO NPs alone, is a consistent finding in the literature for well-designed nanocarrier systems. To quantitatively confirm the interaction between the nanocomposite components, the FICI was determined (Table S4). For the reference strain *P. aeruginosa* ATCC 27853, the FICI was 0.47, indicating a synergistic effect (FICI ≤ 0.5). Most notably, for clinical isolates such as *P. aeruginosa* YMA1, *P. aeruginosa* YMD2, and *P. aeruginosa* YME1, where individual resistance was high, the FICI values were calculated as low as 0.10 and 0.13.

### Ultrastructural study of ZnO NPs- and ZnO/CS/AMX-treated *P. aeruginosa* ATCC 27853

**Fig. 8:**
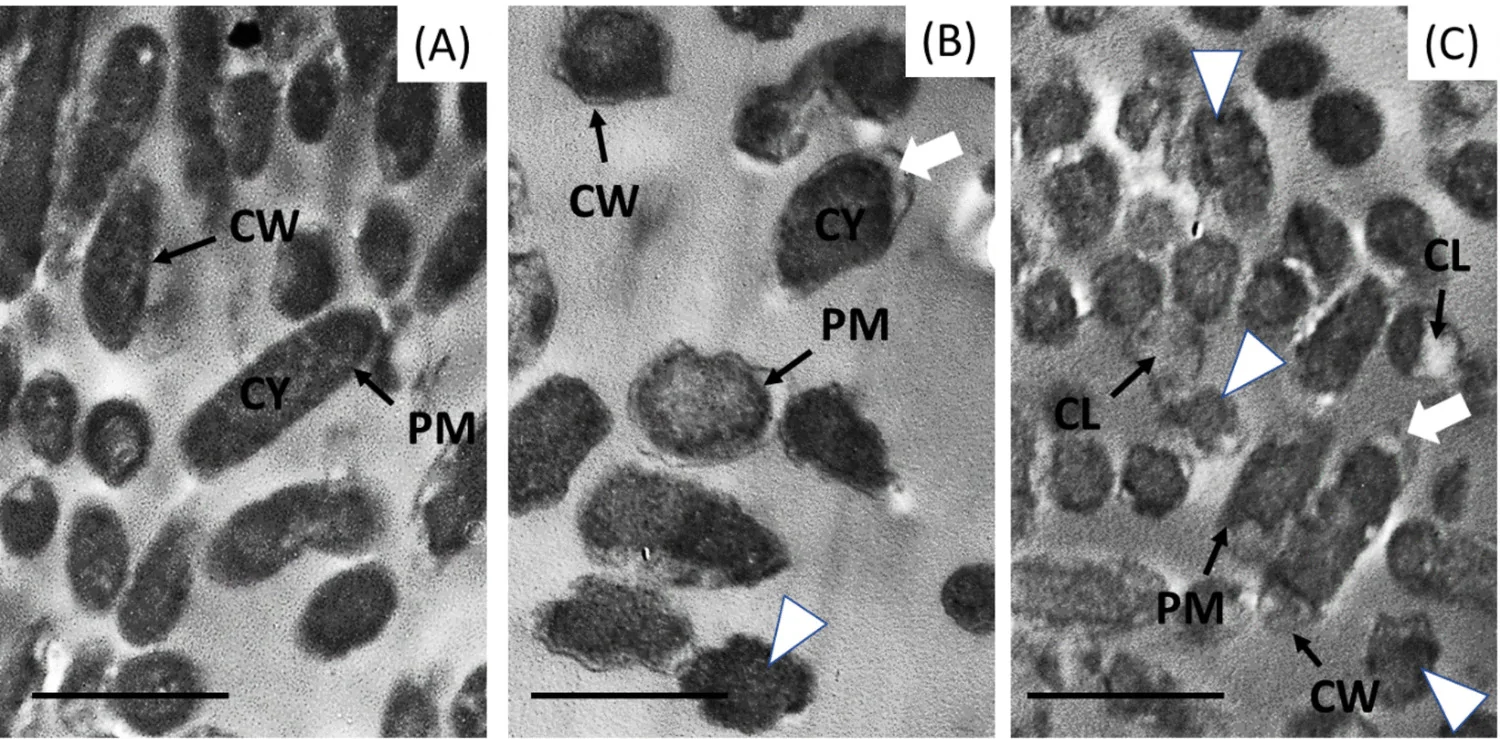
Representative TEM micrographs of *P. aeruginosa* ATCC 27853. **A** Control group: cells appear intact with normal rod shapes, clearly defined cell walls (CW) and plasma membranes (PM). **B** ZnO/CS/AMX-treated (intermediate damage): Cells appear with severe malformation (white arrowhead). Notable separation of the plasma membrane (PM) from the cell wall (CW) and cytoplasmic (CY) condensation (white arrows). **C** ZnO/CS/AMX-treated (severe damage): Extensive cytoplasmic leakage (CL) and complete cell lysis resulting in “ghost cells” (white arrows). Scale bars in all panels represent 1 µm. Observations were based on the examination of multiple grids per treatment group to ensure the reported ultrastructural changes reflect the general population

The antibacterial activity of ZnO NPs and the ZnO/CS/AMX nanocomposite against *P. aeruginosa* ATCC 27853 was examined ultrastructurally using TEM in comparison to untreated bacterial cells (Fig. 8). In the control group, more than 95% of the examined cells remained intact with well-defined gram-negative rod morphology and enclosed a dense and homogeneous cytoplasm (Fig. 8A). The cells maintain their characteristic rod-like shape, with no observable signs of structural damage, lysis, or internal disorganization. This confirms their healthy and viable state before exposure to antimicrobial agents.

In contrast, the TEM micrographs (Fig. 8 B and C) show a systematic pattern of cellular destruction. Figure 8B displays the severe ultrastructural alterations induced in *P. aeruginosa* ATCC 27853 cells after treatment with ZnO NPs. The TEM micrograph demonstrates significant damage, including visible separation between the cell wall and the cytoplasmic membrane. This detachment suggests a disruption of the cell envelope’s integrity. Furthermore, the presence of various degrees of cell lysis is evident, characterized by the rupture of the cell membrane and leakage of intracellular contents. Numerous cells also display severe malformation, appearing distorted and irregular, deviating significantly from the typical rod shape of untreated cells. While cells treated with the ZnO/CS/AMX nanocomposite exhibited extensive damage. Approximately 85% of the observed population showed significant separation between the cell wall and cytoplasmic membrane, with roughly 60% displaying complete cytoplasmic leakage or “ghost cell” formation (Fig. 8C). The level of cellular destruction observed here appears to be more widespread and severe when compared to treatment with ZnO NPs alone.

### Antibiofilm potential of ZnO NPs and ZnO/CS/AMX

**Fig. 9:**
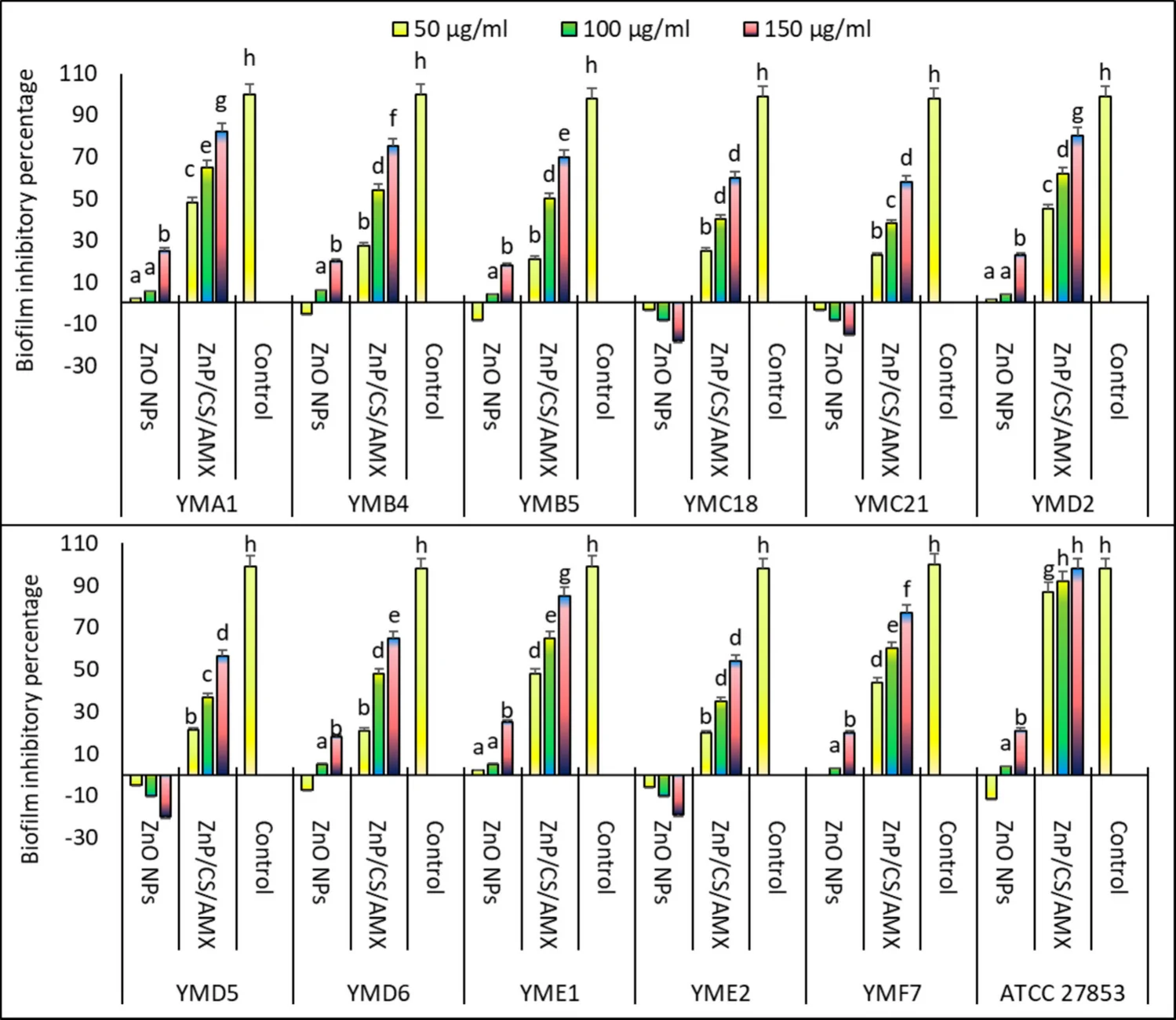
Antibiofilm potential of ZnO NPs and ZnO/CS/AMX in different concentrations (50, 100, and 150 µg/ml) against biofilm-producing bacteria

The antibiofilm potential of ZnO NPs and the ZnO/CS/AMX nanocomposite at varying doses (50, 100, and 150 µg/ml) against biofilm-producing clinical isolates of *P. aeruginosa* and the reference strain ATCC 27853 is illustrated in Fig. 9. The study focuses on the ability of the nanocomposite to interfere with initial bacterial attachment and the early stages of the EPS matrix development. The ZnO NPs alone exhibited a concentration-dependent antibiofilm activity, although this effect was often modest, especially at lower concentrations. At 50 µg/ml, ZnO NPs showed minimal or even slightly negative inhibition for some isolates, suggesting they were not highly effective at this concentration. As the concentration increased to 100 µg/ml and 150 µg/ml, the biofilm inhibitory percentage generally increased for most isolates. For instance, against ATCC 27853, ZnO NPs achieved 4% inhibition at 100 µg/ml and 21% at 150 µg/ml. While showing some activity, the overall antibiofilm efficacy of ZnO NPs as a standalone agent remained comparatively lower than the nanocomposite, particularly against the more robust clinical isolates.

In contrast, the ZnO/CS/AMX consistently demonstrated a significantly superior and concentration-dependent ability to inhibit the biofilm formation of *P. aeruginosa* isolates. Even at the lowest concentration of 50 µg/ml, the nanocomposite showed substantial biofilm inhibition, ranging from 20 to 40% for various isolates. Its efficacy dramatically increased with higher concentrations. At a concentration of 100 µg/ml, the nanocomposite achieved biofilm inhibition percentages typically ranging from 40% to over 60%. At 150 µg/ml, it reached remarkable inhibition levels, often exceeding 80% and reaching nearly 100% for some isolates such as *P. aeruginosa* YMA1, YMD2, YME1, and *P. aeruginosa* ATCC 27853. Specifically, when tested against ATCC 27853, the nanocomposite achieved approximately 98% inhibition at 150 µg/ml, a performance significantly better than that of ZnO NPs alone.

### Cytotoxicity assay

**Fig. 10:**
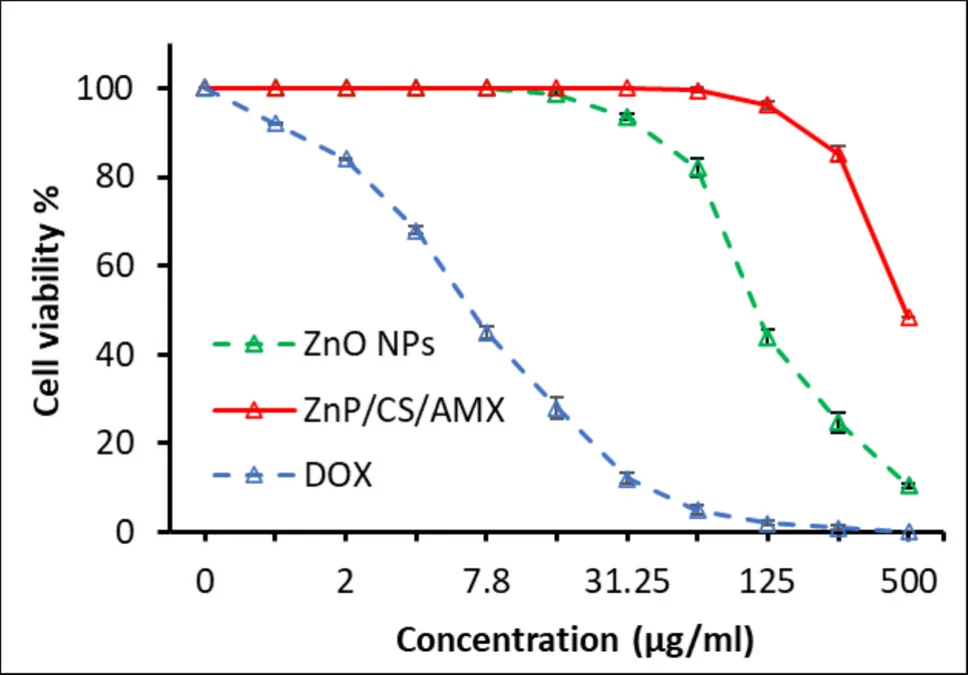
The cytotoxic effects of ZnO NPs (CC_50_ = 106 ± 0.9 µg/ml) and ZnO/CS/AMX (CC_50_ = 292 ± 1.3 µg/ml) compared to the standard DOX (CC_50_ = 6.25 ± 0.7 µg/ml) against Vero cell line

An essential step in determining the biocompatibility of ZnO NPs and the ZnO/CS/AMX nanocomposite for possible biomedical applications is evaluating their in vitro cytotoxicity using a Vero cell line (Fig. 10). The findings unequivocally show that both ZnO NPs and ZnO/CS/AMX have a dose-dependent cytotoxic impact. The DOX has a CC_50_ of 6.25 ± 0.7 µg/ml. However, their toxicity profiles differed significantly and favorably. At doses higher than 106 ± 0.9 µg/ml, ZnO NPs alone began to exhibit a noticeable cytotoxic effect against the Vero cells. While with a CC_50_ of 292 ± 1.3 µg/ml, cell viability decreased drastically as the concentration of ZnO/CS/AMX increased.

To assess the therapeutic safety margin of the ZnO/CS/AMX nanocomposite, the selectivity index (SI) was determined by calculating the ratio of the CC_50_ (292 ± 1.3 µg/ml) to the MICs for the *P. aeruginosa* isolates. As shown in Table S5, the SI values ranged from 7.31 to 29.25. Most importantly, the green synthesis approach yields a highly safe therapeutic profile with a SI of 29.25 and a CC_50_ of 292 ± 1.3 µg/ml (for the reference strain and susceptible clinical isolates; YMA1, YMD2), indicating that it is lethal to bacteria at concentrations nearly 30 times lower than those affecting mammalian cells which provide a safer and more effective alternative to commercial metal-oxide NPs like Ag, Cu, TiO_2_, ZnO, or their combinations (Table S6).

## Discussion

The prevalence of MDR strains is increasing globally, underscoring the pressing demand for novel antimicrobial agents and strategies, such as the ZnO/CS/AMX nanocomposite investigated in this study to overcome established resistance mechanisms and combat this persistent bacterial menace. *P. aeruginosa*, an opportunistic gram-negative bacterium, is recognized as a top-priority pathogen worldwide due to its capacity to form robust biofilms and its intrinsic resistance to multiple classes of treatment (Yang et al. 2025). This pathogen is a significant contributor to serious and often fatal HAIs, including sepsis, ventilator-associated pneumonia, and urinary tract infections (UTIs), particularly in patients with severe burns, cystic fibrosis, or compromised immune systems (de Abreu et al. 2014). According to Gellatly and Hancock (2013), *P. aeruginosa*’s ability to utilize a wide range of virulence factors, such as pyocyanin, elastase, and exotoxin A, complicates therapy and is a major contributor to high rates of morbidity and mortality. Importantly, the bacterium is characterized by the formation of a thick biofilm, which serves as a protective extracellular matrix acting as both a physiological and physical barrier. This makes the embedded bacteria up to 1000 times more resistant to host immune responses and the penetration of traditional antibiotics (Moser et al. 2021).

Eleven *P. aeruginosa* isolates were isolated and species-level identified using the Vitek 2 technique that were labeled as YMA1, YMB4, YMB5, YMC18, YMC21, YMD2, YMD5, YMD6, YME1, YME2, and YMF7.

The AST profiles of the *P. aeruginosa* isolates were evaluated, revealing that strain *P. aeruginosa* YMB5 exhibited a pronounced multidrug-resistant phenotype against the majority of the tested antibiotics. This was followed in order of decreasing resistance by strains *P. aeruginosa* YMC18, *P. aeruginosa* YMC21, and *P. aeruginosa* YMD6. Similarly, OkonK et al. (2009) also found similar results. Shewatatek et al. (2014) and Bilal et al. (2015) reported that the clinical *P. aeruginosa* isolates exhibited 100% resistance to ampicillin. Furthermore, the current study documented that the isolates’ resistance to nitrofurantoin and doxycycline is 90.9%, 90.9%, and 81.8%, respectively. The results indicate that the clinical isolates of *P. aeruginosa* showed multidrug resistance. This broad resistance profile highlights the clinical problem these *P. aeruginosa* strains offer and the need for new therapeutic approaches, especially the universal resistance to penicillin-class antibiotics and the high resistance to others like nitrofurantoin. With resistance to only AMX and ampicillin, isolate YMA1 seems to be the most vulnerable. According to Swetha et al. (2017), *P. aeruginosa* isolates were 100% resistant to ampicillin, penicillin, and oxacillin and were most responsive to vancomycin (5.3%) and tetracycline (10.5%). Abou-Khadra et al. (2024) reported the resistance patterns of various *P. aeruginosa* isolates against several antibiotics. The resistance rates were as follows: doxycycline (83.3%), oxytetracycline (77.8%), ampicillin (83.3%), amoxicillin (88.9%), neomycin (72.2%), and erythromycin (77.8%).

One of the main causes of antibiotic treatment failure and recurrent infections is the phenotypic adaptability to form biofilm (Moore et al. 2016; Reynolds and Kollef 2021). Compared to environmental or acute infection strains, isolates of *P. aeruginosa* from clinical samples, particularly those from chronic infections such as cystic fibrosis, indwelling catheters, or wound infections, have a well-established capacity to form biofilms. *P. aeruginosa* YMD5 has the highest ability of biofilm formation followed by *P. aeruginosa* YMC18, *P. aeruginosa* YMC21, and *P. aeruginosa* YME2. Previous studies have shown that some strains are better able to form biofilms than others (Moskowitz et al. 2004). de Sousa et al. (2023) reported that approximately 92% of the 25 *P. aeruginosa* isolates from UTI samples formed biofilms, with 44% exhibiting low biofilm formation, 24% showing moderate biofilm formation, and 24% displaying high biofilm formation ability. Additionally, Lordelo et al. (2024) examined biofilm formation and surface modification potential in hospital strains of *P. aeruginosa* and *K. pneumoniae*. Most *P. aeruginosa* strains were classified as potent biofilm producers. The study indicated that 21.2% of the 25 *P. aeruginosa* isolates had a moderate propensity for biofilm formation, while 66.7% of the isolates were strong biofilm producers. Furthermore, 9.1% of the *P. aeruginosa* strains showed a limited potential to form biofilm, and 3% of the strains did not show any potential.

The *P. aeruginosa* isolates were screened for the extracellular biosynthesis of ZnO NPs using their cell-free supernatants. The white color of the sample was due to the stimulation of the SPR of the ZnO NPs (Lee et al. 2008). The ZnO NP formation was verified by UV–Vis spectrometric measurement, where absorbance peaks ranging from 340 to 380 nm were observed during the production of ZnO NPs using the cell-free metabolites from various *P. aeruginosa* isolates. The slight variations in peak positions and absorbance values within this narrow range are indicative of subtle differences in NPs size, shape, or degree of aggregation. This suggests that each isolate might have a unique reducing and capping agent profile influencing the final NP characteristics (Singh et al. 2018). ZnO NPs were only produced by cell-free bacterial metabolites and proteins, as evidenced by the sample’s color remaining unchanged in the control investigations. The results showed that *P. aeruginosa* YMC18 had the highest production rate of ZnO NPs followed by *P. aeruginosa* YMD5. The high production yields suggest that YMD5 and YMC18 have superior enzymatic or metabolic capabilities for reducing zinc nitrate precursors into ZnO NPs. This process potentially involves reductase enzymes, organic acids, or proteins secreted into the extracellular medium (Hamedi et al. 2017). These differences highlight the variability in biogenic synthesis capabilities even within the same bacterial species, emphasizing the importance of screening diverse microbial isolates for optimal production of NPs. The identification of YMD5 and YMC18 as high-yield producers is crucial for scaling up the green synthesis process of ZnO NPs. This makes them promising candidates for further investigation and use in the development of the ZnO/CS/AMX nanocomposite (El-Fawal et al. 2024). On the other hand, Abdo et al. (2021) green synthesized ZnO NPs using the *P. aeruginosa* NMJ15*,* resulting in an absorption peak at 380 nm. Barsainya and Singh (2018) also biosynthesized ZnO NPs using *P. aeruginosa* DM1, which exhibited a characteristic absorption peak at 416 nm. The red-shifted and broad peaks are typically associated with contaminants and insufficient reducing agents, leading to NP aggregation (Waychunas 2001; Govindasamy et al. 2024b).

The optimized biosynthesized ZnO NPs produced by *P. aeruginosa* YMC18 were combined with CS and AMX as a new nanocomposite. The prepared nanocomposite was successfully synthesized, its chemical interactions were clarified, and its constituent parts were characterized by FTIR, XRD, TEM, and zeta potential analyses. The FTIR spectrum confirmed the presence of amide I and amide II that might help in increasing of the nanocomposite stability (Fanelli et al. 2018). The successful synthesis of ZnO NPs was confirmed by the presence of metal NPs at 400–700 cm^−1^ (Ashokkumar and Muthukumaran 2014). A prominent and broad absorption band typically observed below 600 cm^−1^, especially around 450 cm^−1^, defines the spectra of ZnO NPs. This peak corresponds to the characteristic stretching vibration of the Zn–O bond, confirming the presence of zinc oxide (Gomaa 2022; Fayed et al. 2024). Several alterations were noted in the FTIR spectrum of the ZnO/CS/AMX nanocomposite, particularly the shifts and reduced intensity of key AMX and CS functional groups, indicating molecular interactions—likely hydrogen bonding or electrostatic forces—among the amoxicillin medication, the chitosan polymer, and the ZnO NPs’ surface (Attia et al. 2022; Jasem and Mahmood 2023). The efficient incorporation of AMX into the ZnO/CS matrix without significant chemical degradation is confirmed by the presence of all characteristic peaks, albeit with some changes. Therefore, the overall spectrum of the nanocomposite provides strong spectroscopic evidence of the successful production of the ternary composite material, indicating a stable entrapment or conjugation of AMX onto the CS-coated ZnO NPs.

The XRD pattern of ZnO NPs was studied and displayed several sharp diffraction peaks. The sharpness and narrowness of these peaks further imply that the biosynthesized ZnO NPs are well-formed and of good crystalline quality (Mohan and Renjanadevi 2016). The great purity of the produced ZnO NPs with no discernible crystalline imperfections is indicated by the absence of any extra peaks. According to the XRD pattern of ZnO/CS/AMX, instead of just physical mixing where distinct ZnO peaks would remain with high intensity, the successful incorporation of ZnO NPs into the polymeric/drug matrix is confirmed by this decrease in crystallinity and the masking effect. The absence of new crystalline peaks that could be attributed to CS or AMX provides further evidence that they are either in an amorphous state or have high dispersion, rendering XRD detection impossible.

Controlling the release kinetics of AMX and providing a stable platform for the nanocomposite’s antibacterial activity rely on this integration. The FTIR and XRD results, which show that the organic components greatly affect the spectroscopic fingerprints of ZnO, are consistent with this visible proof of encapsulation. TEM and zeta potential measurements were also used to examine the morphological properties, particle size distribution, and colloidal stability of the produced ZnO/CS/AMX nanocomposite. The dark areas indicate the presence of the higher electron-density ZnO core, which appears to be evenly distributed within a lighter, more amorphous matrix. This matrix likely corresponds to the encapsulated AMX and CS polymer (Nagpal 2024). The 100-nm scale bar confirms that the individual composite particles are indeed within the nanoscale range. Several aspects of green synthesis techniques, such as differences in the concentrations of reducing/capping agents and reaction rates, may be responsible for this broadness and possible multimodality (Duan et al. 2015). Because the polymer layer increases the ZnO core’s physical size, the presence of CS also affects the hydrodynamic diameter (El-Zahed et al. 2024a).

ZnO/CS/AMX exhibited a drug loading of 55.7%. This impressive drug loading efficiency is a significant finding, indicating that the synthesized CS-coated ZnO NPs serve a valuable substrate for attaching or trapping a substantial quantity of AMX. In their study, Jasem and Mahmood (2023) found that the formulation with a 1:2 ratio of AMX-CS NPs exhibited a maximum loading efficiency of 53 ± 2.4%. This high drug loading percentage is beneficial for therapeutic purposes because it means that a smaller amount of the nanocomposite material would be needed to administer a therapeutically effective dose of AMX. This not only enhances the potential efficacy of the antimicrobial agent but also potentially reduces the overall dosage of the NPs carrier itself, minimizing any potential cytotoxic effects (Mondal et al. 2023; Mamidi et al. 2025).

The zeta potential of the ZnO/CS/AMX nanocomposite was recorded to have a net positive surface charge (+ 35 ± 2.3 mV). The main cause of this positive charge is CS, a cationic polymer with several -NH_2_ groups that protonate in acidic to neutral aqueous solutions (Samal et al. 2012). Antimicrobial applications greatly benefit from the positive zeta potential. The membranes of many bacteria, including *P. aeruginosa*, have a net negative charge because of substances like lipopolysaccharides. Therefore, it is expected that a positively charged nanocomposite will exhibit enhanced electrostatic attraction to negatively charged bacterial surfaces, facilitating adhesion, membrane breakdown, and subsequent antimicrobial activity (Alfei and Schito 2020). Furthermore, a high absolute zeta potential value (usually exceeding 30 mV) generally signifies good colloidal stability. This suggests that the ZnO/CS/AMX nanocomposite remains stable against agglomeration in suspension, crucial for maintaining its nanoscale properties and bioavailability across various applications (Wang and Keller 2009). The combination of size and positive surface charge makes this an advantageous choice for anticipated anti-*P. aeruginosa* action. Conversely, *Corynebacterium* sp. produced ZnO NPs with a positive charge of approximately + 18.9 mV, a size range of 8 to 17 ± 1.23 nm, and a spherical shape (Abd El-Nour et al. 2023). According to Ahmad Yusof et al. (2019), ZnO/CS was produced with a uniform distribution, averaging 70 nm and possessing a spherical shape. According to Attia et al. (2022), the Zn ONPs’ zeta potential was −24.7 mV, with an average particle size of 7.99 nm.

The anti-*Pseudomonas* action of ZnO/CS/AMX was studied compared to ZnO NPs and imipenem. All tested *P. aeruginosa* clinical isolates demonstrated complete resistance to CS and AMX when administered as individual agents alone (data not shown). This inherent resistance to the individual components underscores the challenge in treating these isolates and highlights the potential significance of using a nanocomposite treatment. This aligns with the global concern regarding increasing carbapenem resistance in *P. aeruginosa* clinical isolates (Lister et al. 2009). On the other hand, ZnO NPs and ZnO/CS/AMX exhibited better antibacterial activity against all tested strains. Several studies have reported inhibition zones of 14 mm to 25 mm against *P. aeruginosa* (Dhanasegaran et al. 2021; El-Fallal et al. 2023; HajiSadeghi et al. 2025). However, others have shown bacterial resistance to ZnO NPs (Wang et al. 2018; Gudkov et al. 2021). Additionally, Zhang et al. (2018) reported that some bacteria such as *E. coli* have the ability to develop adaptive resistance to ZnO NPs after days of exposure. Previous studies have reported that the presence of CS enhances the antibacterial action of nanocomposites (El-Fawal et al. 2024; El-Zahed et al. 2024a; Elshikiby et al. 2025).

The remarkable decrease in MIC/MBC confirms the strong bactericidal synergy of the composite system. The FICI values fall well below the 0.5 threshold, mathematically proving a potent synergistic interaction that restores antimicrobial efficacy against resistant strains. The nanocomposite showed a 15-fold reduction in the MIC compared to ZnO NPs alone for isolate *P. aeruginosa* YMA1, resulting in an FICI of 0.07, confirming a high degree of synergistic potentiation. Similar synergistic effects have been reported for CS-coated metallic NPs combined with antibiotics, where the polymer aids in drug delivery and membrane permeabilization, enhancing the action of the loaded antibiotic and the NPs themselves (Masoumeh et al. 2020; Khan et al. 2022b). The ability of the nanocomposite to achieve bactericidal effects at significantly lower concentrations is highly important for clinical translation, as it suggests the potential to eradicate *P. aeruginosa* infections more effectively while potentially minimizing off-target toxicity by reducing the required dose of individual components. Previous studies have shown that biosynthesized ZnO NPs and their nanocomposites typically have MIC values against *P. aeruginosa* ranging from 64 to > 256 µg/ml, depending on particle size, synthesis method, and the specific bacterial strain (Shome et al. 2023; HajiSadeghi et al. 2025). The observed MIC values for ZnO NPs alone and ZnO/CS/AMX ranged from 10 µg/ml to as high as 150 µg/ml for certain resistant isolates (YMA1, YMD2). These values fall within or slightly below this reported range, indicating comparable inherent activity. Furthermore, studies on CS as an antibacterial agent against *P. aeruginosa* typically report MIC values upwards of 500 µg/ml to several mg/ml (Tin et al. 2009; Kaur et al. 2015; Sahariah 2016). These studies often highlight its limited efficacy as a standalone agent at lower concentrations, which aligns with our preliminary finding that individual CS was ineffective. For AMX, while *P. aeruginosa* is generally less susceptible than gram-positive bacteria, reported MICs for susceptible strains can range from 16 to 128 µg/ml (Ahmad et al. 2025; Seukep et al. 2025). However, the high resistance observed in our clinical isolates to AMX alone further underscores the challenge.

Previous bacterial micrographs have shown that ZnO NPs and their nanocomposites have potent bactericidal effects on cell walls and membranes. This results in the autolysis of most cell organelles and the presence of severely injured cells (El-Fallal et al. 2023; El‑Zahed et al. 2024b). The increased activity can be attributed to a multimodal mechanism: the positively charged CS component facilitates a strong electrostatic attraction to the negatively charged *P. aeruginosa* cell membrane, allowing for the effective delivery of both the ZnO NPs and the AMX payload (El-Araby et al. 2024). This increased permeation allows the ZnO NPs to exert their membrane-disrupting and oxidative stress-inducing effects, while simultaneously delivering AMX, which can then inhibit cell wall synthesis (Gibson and Veening 2023). The physical disruption caused by ZnO NPs and their interaction with the CS membrane likely compromise bacterial efflux pumps and membrane integrity. This renders the cells more vulnerable to AMX, effectively bypassing the pre-existing resistance mechanisms to AMX alone. This multi-pronged attack strategy is highly effective against adaptable pathogens like *P. aeruginosa*, offering a promising therapeutic approach, especially for strains that exhibit resistance to traditional antibiotics and individual antimicrobial agents. The product transforms a failed antibiotic, AMX, into a lethal weapon against MDR strains by using the ZnO/CS matrix as a mechanical drill to penetrate the cell wall, providing a pathway that traditional chemical resistance cannot easily block. Other mechanisms of ZnO NPs are also associated with the production of ROS (superoxide anions, hydroxyl radicals, and hydrogen peroxide), interaction with the cell barrier (cell-wall rupture and permeability change), suppression of protein and DNA synthesis, obstruction of bacterial enzymatic activities, metabolic gene expression, and other aspects (Madkour 2020; Zhang et al. 2021). Future studies should investigate these important impacts because ROS can damage bacterial cell membranes, thereby inhibiting DNA and amino acid replication.

Biofilms are famously difficult to eliminate and are often much more resistant to antibiotics than planktonic cells due to their protective EPS matrix and the altered physiological state of embedded bacteria (Donlan and Costerton 2002). A study by Dey et al. (2020) examined how various antibiotics affected *P. aeruginosa*’s ability to produce biofilms using the crystal violet method. They discovered that the same antibiotics were more effective than others in preventing biofilms. Although ZnO NPs can inhibit the formation of bacterial biofilms, their ability to penetrate and effectively inhibit established or forming biofilms may be limited due to the protective EPS matrix (Alhosani et al. 2025; Afsharikhah et al. 2025; Fattahi et al. 2025). Numerous studies have reported the antibiofilm activity of ZnO NPs against *P. aeruginosa*. Inhibition rates vary widely depending on synthesis methods, particle characteristics, and concentrations. For example, ZnO NPs synthesized via green routes have been shown to inhibit *P. aeruginosa* biofilms by 30–70% at concentrations ranging from 100 to 500 µg/ml. This is primarily achieved through the generation of ROS and disruption of the EPS matrix (Al-Momani et al. 2024; Konkuri et al. 2024). The current findings for ZnO NPs alone show up to 25% inhibition at 150 µg/ml for *P. aeruginosa* ATCC 27853 and similar modest effects for clinical isolates. These results are consistent with previous reports, suggesting that while effective, standalone ZnO NPs may have limitations against more resistant biofilms.

A dramatically enhanced antibiofilm activity of the ZnO/CS/AMX nanocomposite can be attributed to the synergistic interplay of its components. This effect is attributed to the anti-adhesive properties of the positively charged CS coating. It physically prevents bacterial docking on surfaces, thereby facilitating deeper penetration of both ZnO NPs and AMX into the biofilm structure (Donlan and Costerton 2002). The combined effect of ZnO-induced ROS, along with AMX’s inhibition of cell wall synthesis, could degrade early-stage EPS components before a robust, mature biofilm can be established. By inhibiting the initial stages of biofilm development, the nanocomposite prevents the transition from planktonic to sessile growth, which is a critical step in chronic *P. aeruginosa* infections. CS-coated ZnO or Ag NPs have been reported to have a superior antibiofilm activity compared to bare NPs, with recorded biofilm reductions of 60–90% against *P. aeruginosa* at concentrations typically above 100 µg/ml (Badawy et al. 2020; Hemmati et al. 2020; Thirupathi et al. 2022). Furthermore, the encapsulation of antibiotics into NPs or polymeric carriers has proven to be a successful strategy in overcoming biofilm-mediated resistance. Studies have shown that AMX-loaded polymeric NPs can significantly reduce *P. aeruginosa* biofilms at concentrations where free AMX is ineffective, thanks to the protection provided by the biofilm (Khoshnood et al. 2023; Chand and Kushawaha 2024a, 2024b). More research is needed to fully understand the mechanism of action of ZnO NPs and ZnO/CS/AMX in eliminating bacterial biofilms.

The level of toxicity for uncoated ZnO NPs was studied against Vero cells and was consistent with numerous reports in the literature indicating that concentrations above 50–100 µg/ml often result in cytotoxicity in various mammalian cell lines. This is primarily due to the release of toxic Zn^2+^ ions, membrane damage, and the production of ROS (Abdel-Aziz et al. 2019; Abdelrahman et al. 2023). In contrast, ZnO/CS/AMX exhibited significantly lower cytotoxicity, indicating a more favorable safety profile. Up to doses of 125 µg/ml, which is significantly greater than the onset of toxicity for bare ZnO NPs, the nanocomposite preserved almost 100% cell viability. With a CC_50_ of 292 ± 1.3 µg/ml, cell viability only started to decline noticeably at concentrations higher than 125 µg/ml. Compared to ZnO NPs, the CC_50_ value of this nanocomposite is about three times higher, indicating that the nanocomposite is significantly less harmful to mammalian cells. This significant decrease in cytotoxicity for the nanocomposite is an important discovery, likely due to the CS coating. As a biocompatible and biodegradable polymer, CS acts as a barrier that can reduce the direct interaction of the ZnO core with the cell membrane, decrease the release of cytotoxic Zn^2+^ ions, and lower the production of excessive ROS by host cells (Dutta et al. 2012; Divya et al. 2017; Sun et al. 2022). Because it allows for the use of higher concentrations to achieve effective antibacterial and antibiofilm activity while staying below cytotoxic levels for mammalian cells, this improved biocompatibility expands the therapeutic range for the nanocomposite (Govindasamy et al. 2021). The computed CC_50_ of the ZnO/CS/AMX nanocomposite is 292 ± 1.3 µg/ml, which is significantly higher than the effective antibiofilm concentrations (as low as 50 µg/ml for substantial suppression) and MIC/MBC values (as low as 10–15 µg/ml) found against *P. aeruginosa*. Even for isolates with higher MICs, the SI remained significantly high, suggesting a favorable therapeutic window for further preclinical investigations. While the ZnO/CS/AMX nanocomposite demonstrated low cytotoxicity against the Vero cells, it is important to acknowledge the limitations of these in vitro findings. The use of a single mammalian cell line offers only a preliminary assessment of biocompatibility. Therefore, while the high selectivity indices are promising, further in vivo toxicological studies and evaluations against multiple human cell types are necessary to fully establish the clinical safety profile and therapeutic window of this system.

## Conclusions

This study successfully developed and extensively characterized a novel ZnO/CS/AMX nanocomposite as a potent and biocompatible antimicrobial agent against challenging *Pseudomonas aeruginosa* infections. Our findings demonstrate a compelling synergistic effect, where the composite’s efficacy significantly surpasses that of its individual components and often rivals or exceeds that of conventional antibiotics. The detailed antimicrobial assays first revealed that the ZnO/CS/AMX nanocomposite consistently achieved remarkably low MICs and MBCs against a panel of *P. aeruginosa* clinical isolates that were inherently resistant to AMX and CS alone. This potent bactericidal action was visually confirmed by TEM, which showed extensive cellular damage, including membrane disruption, cell lysis, and severe morphological alterations in treated bacterial cells. Secondly, the nanocomposite exhibited outstanding antibiofilm capabilities, showing a dose-dependent effect that resulted in nearly 100% inhibition of *P. aeruginosa* biofilm formation at therapeutic concentrations. This is a crucial advantage, considering the well-known resistance of biofilms to traditional antimicrobial treatments. The multimodal action, which likely includes chitosan’s ability to permeabilize membranes and disrupt EPS, along with ZnO’s induction of oxidative stress and AMX’s inhibition of cell wall formation, effectively combats the intricate protective mechanisms of *P. aeruginosa* biofilms. Finally, and crucially for clinical translation, the ZnO/CS/AMX nanocomposite demonstrated a wide therapeutic window in a cellular model compared to bare ZnO NPs, with a significantly higher CC_50_ in Vero cell lines. This favorable biocompatibility ensures a broad therapeutic window, enabling the effective eradication of bacterial pathogens and biofilms at concentrations well below those toxic to host cells. In conclusion, the novel ZnO/CS/AMX represents a highly promising and safe therapeutic platform. Its potent synergistic antimicrobial and antibiofilm activities, coupled with its promising biocompatibility, position it as an innovative solution for tackling multidrug-resistant and biofilm-associated *P. aeruginosa* infections, addressing an urgent unmet need in clinical antimicrobial therapy. Future studies will focus on in vivo efficacy and pharmacokinetics to further validate its clinical potential.

## Supplementary Information

Below is the link to the electronic supplementary material.

ESM 1 (DOCX 329 KB)

## Data Availability

Upon reasonable request, the corresponding author will provide the datasets generated during the current work. Upon reasonable request, the relevant author will make the datasets used and/or analyzed in the current work available.
