# Supplementary material for: Optimized green zinc oxide/chitosan/amoxicillin nanocomposite against Pseudomonas aeruginosa
Source: Appl Microbiol Biotechnol. 2026 Mar 24;110(1):119. doi: 10.1007/s00253-026-13768-3 (PMC13018007; doi:10.1007/s00253-026-13768-3)
Supplement: Supplementary file 1 — (DOCX 329 KB) [file 253_2026_13768_MOESM1_ESM.docx]

**Optimized Green Zinc Oxide/Chitosan/Amoxicillin Nanocomposite against *Pseudomonas aeruginosa***

Mohamed M. El-Zahed*, Yassmein M. Radwan, Mohamed I. Abou-Dobara

Department of Botany and Microbiology, Faculty of Science, Damietta University, New Damietta, 34517, Egypt

^*^Correspondence email: [mohamed.marzouq91@du.edu.eg](mailto:mohamed.marzouq91@du.edu.eg)

^*^ORCID: 0000-0003-2694-3720

**Supplementary Tables**

**Table S1.** The various variables and their levels were used to optimize the biosynthesis of ZnO NPs biosynthesis by *P. aeruginosa*.

| **Factor** | **Name** | **Coded level (-1)** | **Coded level (0)** | **Coded level (+1)** |
| --- | --- | --- | --- | --- |
| ***X*_1_​** | Temperature (∘C) | 10 | 30 | 60 |
| ***X*_2_​** | pH | 4 | 7 | 10 |
| ***X*_3_​** | Concentration (%) | 10 | 30 | 100 |
| ***X*_4_​** | Ratio (v/v%) | 1:1 | 1:4 | 1:16 |

**Table S2.** Biochemical tests for *P. aeruginosa* isolates.

| **Biochemical test** | **YMA1** | **YMB4** | **YMB5** | **YMC18** | **YMC21** | **YMD2** | **YMD5** | **YMD6** | **YME1** | **YME2** | **YMF7** |
| --- | --- | --- | --- | --- | --- | --- | --- | --- | --- | --- | --- |
| Catalase | +^*^ | + | + | + | + | + | + | + | + | + | + |
| Citrate utilization | + | + | + | + | + | + | + | + | + | + | + |
| H_2_S production | + | - | - | - | - | - | + | + | + | - | - |
| Indole production | - | - | - | - | - | - | - | - | - | - | - |
| Lipase | + | - | + | + | + | + | + | + | - | - | + |
| Methyl red | - | - | - | - | - | - | - | - | - | - | - |
| Nitrate reduction | + | + | + | + | + | + | + | + | + | + | + |
| Oxidase | + | + | + | + | + | + | + | + | + | + | + |
| Starch fermentation | - | - | - | - | - | - | - | - | - | - | - |
| Urease | - | - | - | + | - | - | + | - | - | - | + |
| Voges-Proskauer | - | - | - | - | - | - | - | - | - | - | - |
| Arabinose fermentation | - | - | - | - | - | - | - | - | - | - | - |
| Glucose fermentation | - | - | - | - | - | - | - | - | - | - | - |
| Gas from glucose | - | - | - | - | - | - | - | - | - | - | - |
| Lactose fermentation | - | - | - | - | - | - | - | - | - | - | - |
| Maltose fermentation | - | - | - | - | - | - | - | - | - | - | - |
| Mannitol fermentation | + | + | + | + | - | + | + | - | + | - | + |

^*^+ = Present; - = Absent.

**Table S3.** ANOVA and model performance of full quadratic model.

| **Source** | **Sum of Squares** | ***df*** | **Mean Square** | **F-Value** | ***p*-Value** | **Significance** |
| --- | --- | --- | --- | --- | --- | --- |
| **Model** | 12.564 | 14 | 0.897 | 3.01 | 0.0384 | Significant |
| **Linear terms** | 0.037 | 4 | 0.009 | 0.03 | 0.999 | Not significant |
| **Quadratic terms** | 12.046 | 4 | 3.012 | 10.09 | 0.0006 | Significant |
| **Interaction terms** | 0.481 | 6 | 0.08 | 0.27 | 0.943 | Not significant |
| **Residual** | 3.582 | 12 | 0.298 |  |  |  |
| **Lack of fit** | 0.111 | 10 | 0.011 | 0.04 | 0.9997 | Not significant |
| **Pure error** | 3.471 | 2 | 1.736 |  |  |  |
| **Cor Total** | 16.146 | 26 |  |  |  |  |

*df* = Degree of Freedmen, F = Fisher-ratio.

**Table S4:** Fractional inhibitory concentration index (FICI) calculation for key isolates

| **Bacterial strain** | **MIC (μg/ml)** | | | **FICI** | **Interpretation** |
| --- | --- | --- | --- | --- | --- |
|  | **ZnO NPs** | **AMX** | **ZnO/CS/AMX** |  |  |
| **ATCC 27853** | 25 | 150 | 10 | 0.47 | Synergy |
| **YMA1** | 150 | >300* | 10 | <0.10 | Strong synergy |
| **YMD2** | 130 | >300* | 10 | <0.13 | Strong synergy |
| **YME1** | >300* | >300* | 20 | <0.13 | Strong synergy |

*Note: Calculation based on a conservative 300 µg/ml threshold for resistant strains.

**Table S5:** Selectivity index (SI) calculation and comparative safety/efficacy

| **Bacterial Strain** | **MIC (µg/ml)** | | **CC_50​_ (µg/ml)** | **SI** | |
| --- | --- | --- | --- | --- | --- |
|  | **ZnO NPs** | **ZnO/CS/AMX** |  | **ZnO NPs** | **ZnO/CS/AMX** |
| **ATCC 27853** | 25 | 10 | 292.45 | 11.69 | 29.25 |
| **YMA1** | 150 | 10 | 292.45 | 1.95 | 29.25 |
| **YMD2** | 130 | 10 | 292.45 | 2.25 | 29.25 |
| **YME1 / YME2** | >300* | 20 | 292.45 | ˂1 | 14.62 |
| **YMB5 / YMC21** | >300* | 30 | 292.45 | ˂1 | 9.75 |
| **YMB4 / YMC18** | >300* | 40 | 292.45 | ˂1 | 7.31 |

*Note: Calculation based on a conservative 300 µg/ml threshold for resistant strains.

**Table S6:** Comparative analysis of commercial nanoparticles and the ZnO/CS/AMX composite

| **Nanoparticle Type** | **Primary strengths** | **Major limitations** | **Clinical relevance/performance** | **References** |
| --- | --- | --- | --- | --- |
| **Ag** | Potent broad-spectrum antibacterial activity. | High toxicity to human cells (low SI); potential for tissue staining (argyria). | Strong, but safety concerns limit systemic use. | (Kim et al. 2008; Khan et al. 2019) |
| **TiO₂** | Chemically stable; inexpensive. | Primarily active under UV light; limited efficacy in the dark. | Reduced effectiveness in physiological environments. | (Govindasamy et al. 2024) |
| **Cu/CuO** | Low cost; high availability. | Rapid oxidation; significant toxicity to mammalian liver/kidney cells. | Safety window is often too narrow for clinical use. | (Govindasamy et al. 2022) |
| **ZnO** | Biocompatible; "GRAS" status; high ROS generation. | Propensity for agglomeration in biological fluids. | Moderate; requires surface modification for stability. | (Bhunia et al. 2016; Asokan et al. 2025) |
| **ZnO/TiO₂ & CuO/TiO₂** | Enhanced photocatalytic activity. | Still highly dependent on light activation for maximum lethality. | Limited application in internal infections. | (Bui et al. 2017; Eymard-Vernain et al. 2020) |
| **ZnO/CuO** | Synergistic metal ion release. | Cumulative toxicity of two metal species can be high. | Risk of heavy metal accumulation. | (Govindasamy et al. 2021; Govindasamy et al. 2023) |
| **Fabricated ZnO/CS/AMX** | Triple synergy (FICI < 0.5); mechanical lysis; high cationic stability (+37.2 mV). | Requires multi-step green synthesis. | Superior: SI of 29.25 and 100% efficacy against MDR *P. aeruginosa*. | Current study |

*Note: SI=selectivity index, FICI=fractional inhibitory concentration index, GRAS=Generally Regarded as Safe, ROS=reactive oxygen species, MDR=multidrug resistant.

**Supplementary Figures**


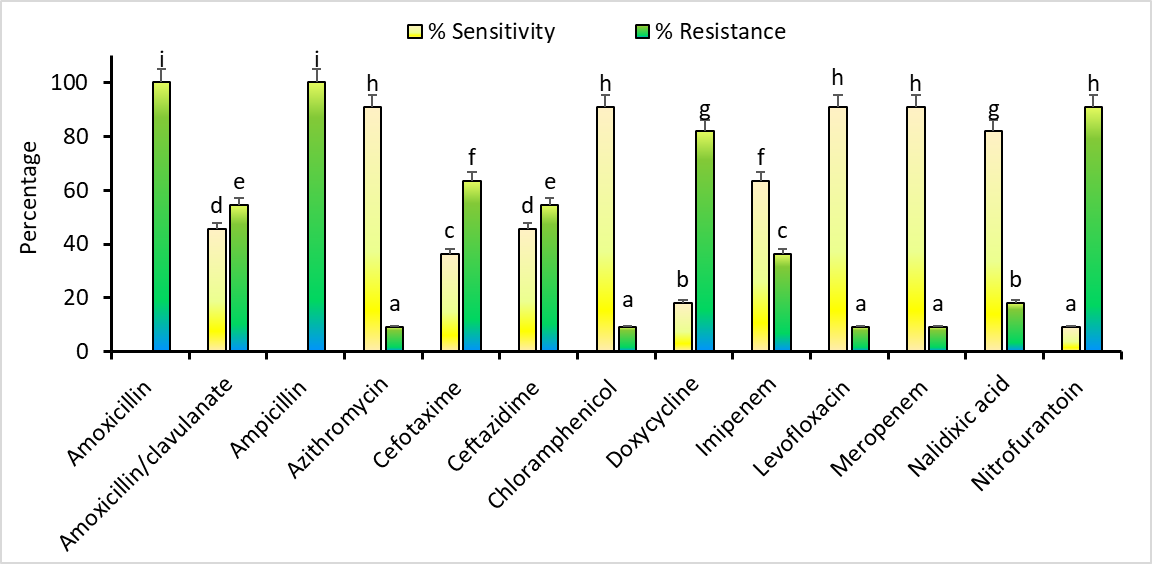


**Fig. S1** Patterns of antibiotic resistance and sensitivity in *P. aeruginosa* isolates. Bars is the antibiotics susceptibility percentage; 0% Sensitivity or 100% Resistance = Absence of *P. aeruginosa* isolates sensitivity. Error bars with common letters are not significantly different according to LSD test (*P* < 0.05).


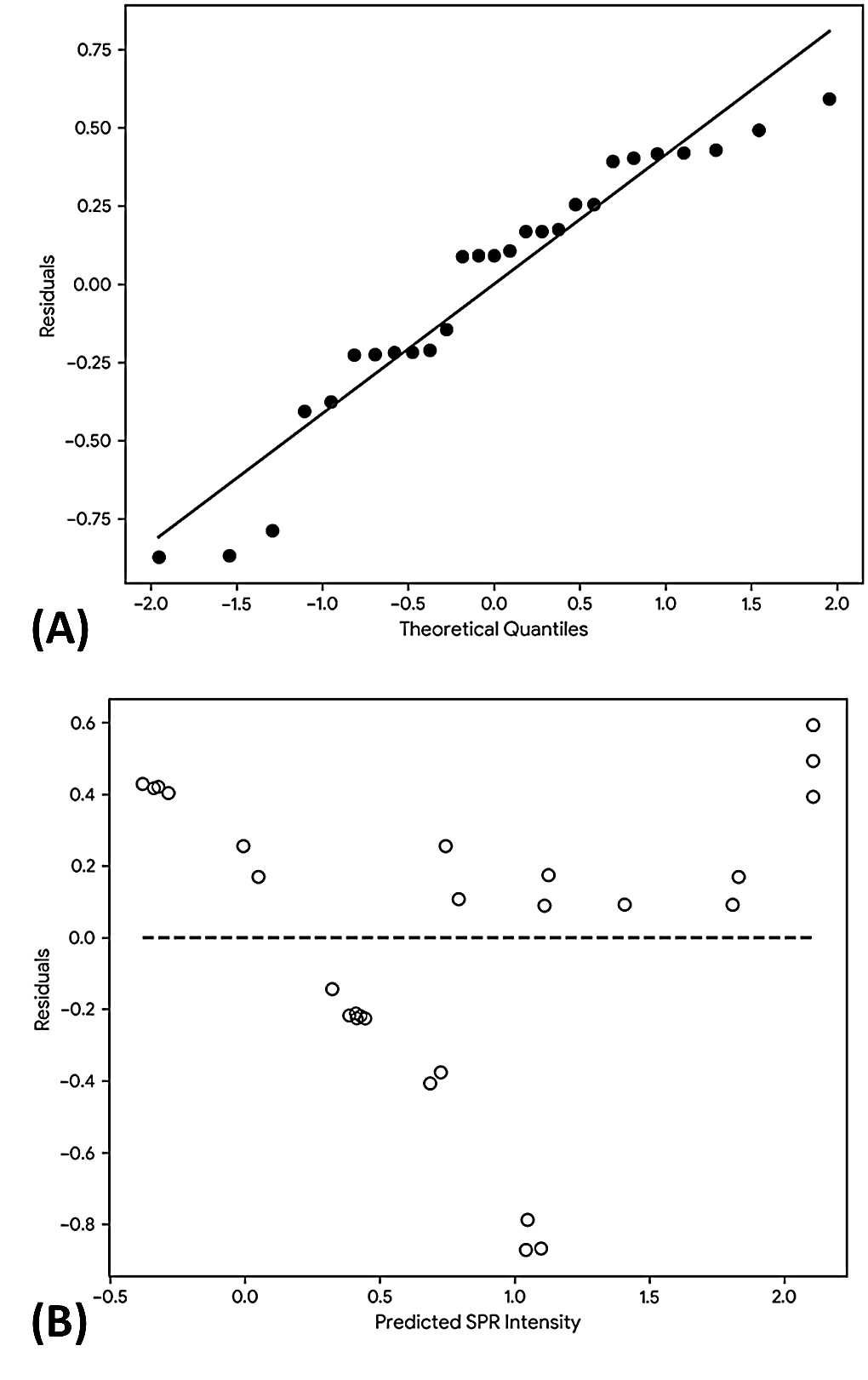


**Fig. S2** (A) Normal probability plot of internally studentized residuals for the quadratic model for SPR intensity. (B) Plot of residual versus predicted SPR intensity.


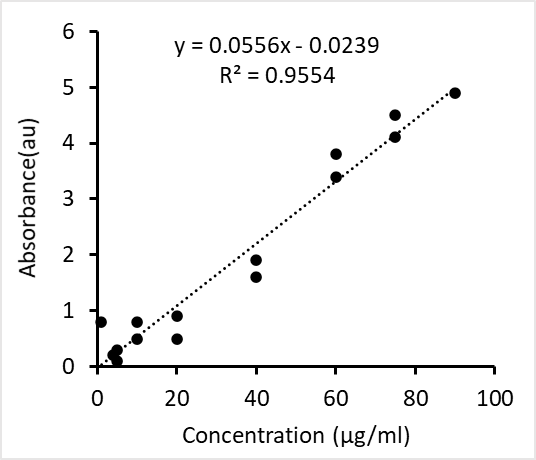


**Fig. S3** Standard curve of AMX in pure water.


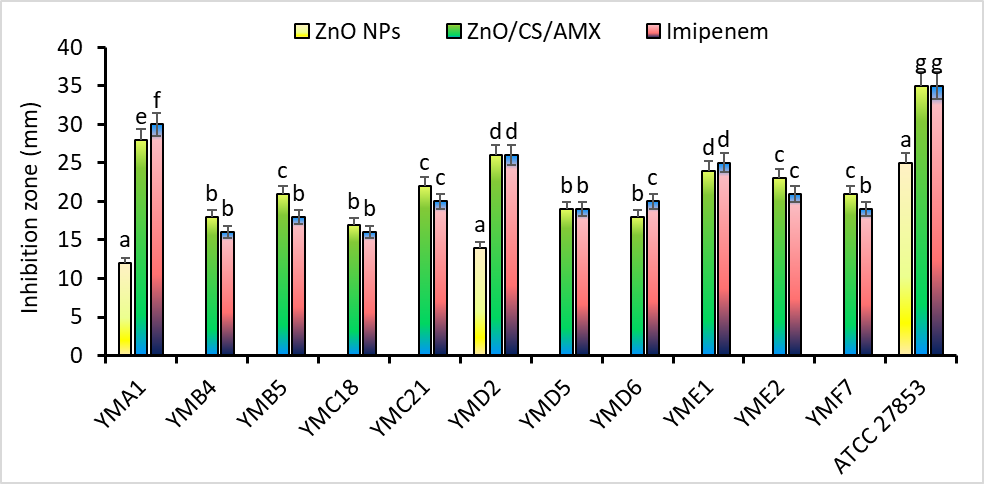


**Fig. S4** Antibacterial activity of ZnO NPs, ZnO/CS/AMX, and imipenem using agar well diffusion method against the different *P. aeruginosa* isolates as well as *P. aeruginosa* ATCC 27853. Error bars with common letters are not significantly different according to LSD test (*P* < 0.05).

**References**

Asokan S, S S, Jacob T, Jisha MS, Vijayan S (2025) Biogenic ZnO nanoparticle-coated endotracheal tubes: A study on their biocompatibility and antibacterial activity. Next Nanotechnol 8:100274. <https://doi.org/10.1016/J.NXNANO.2025.100274>

Bhunia A, Kamilya T, Saha S (2016) Optical and structural properties of protein capped ZnO nanoparticles and its antimicrobial activity. J Adv Biol Biotechnol 10:1–9. <https://doi.org/10.9734/JABB/2016/29626>

Bui VK, Park D, Lee Y-C (2017) Chitosan combined with ZnO, TiO_2_ and Ag nanoparticles for antimicrobial wound healing applications: A Mini review of the research trends. Polymers (Basel) 9:491. <https://doi.org/10.3390/polym9100491>

Eymard-Vernain E, Luche S, Rabilloud T, Lelong C (2020) ZnO and TiO_2_ nanoparticles alter the ability of *Bacillus subtilis* to fight against a stress. PLoS One 15:e0240510. [https://doi.org/10.1371/journal.pone.0240510](https://www.google.com/search?q=https://doi.org/10.1371/journal.pone.0240510)

Govindasamy GA, Mydin RBSMN, Sreekantan S, Harun NH (2021) Compositions and antimicrobial properties of binary ZnO–CuO nanocomposites encapsulated calcium and carbon from *Calotropis gigantea* targeted for skin pathogens. Sci Rep 11:99. <https://doi.org/10.1038/s41598-020-79547-w>

Govindasamy GA, Rabiatul RB, Harun NH, Effendy WNFWE, Sreekantan S (2022) Giant milkweed plant-based copper oxide nanoparticles for wound dressing application: physicochemical, bactericidal and cytocompatibility profiles. Chem Pap 77:1181–1200. <https://doi.org/10.1007/S11696-022-02513-5>

Govindasamy GA, Rabiatul RB, Gadaime NKR, Sreekantan S (2023) Phytochemicals, biodegradation, cytocompatibility and wound healing profiles of chitosan film embedded green synthesized antibacterial ZnO/CuO nanocomposite. J Polym Enviro 31:4393–4409. <https://doi.org/10.1007/S10924-023-02902-1>

Govindasamy GA, Sreekantan S, Saharudin KA, Ong MT, Thavamany PJ, Sahgal G, Tan AA (2024) Effect of compositions and heat treatments of polypropylene/PP-g-MAH/CuO-TiO_2_ composites on thermal, crystallization and antimicrobial properties. BioNanoScience 14:2678–2690. <https://doi.org/10.1007/S12668-024-01453-6>

Khan I, Saeed K, Khan I (2019) Nanoparticles: Properties, applications and toxicities. Arab J Chem 12:908–931. <https://doi.org/10.1016/j.arabjc.2017.05.011>

Kim YS, Kim JS, Cho HS, Rha DS, Kim JM, Park JD, Choi BS, Lim R, Chang HK, Chung YH (2008) Twenty-eight-day oral toxicity, genotoxicity, and gender-related tissue distribution of silver nanoparticles in Sprague-Dawley rats. Inhal Toxicol 20:575–583. [https://doi.org/10.1080/08958370801905464](https://www.google.com/search?q=https://doi.org/10.1080/08958370801905464)
